# Origin and Perspectives of the Photochemical Activity of Nanoporous Carbons

**DOI:** 10.1002/advs.201800293

**Published:** 2018-06-20

**Authors:** Teresa J. Bandosz, Conchi O. Ania

**Affiliations:** ^1^ Department of Chemistry and Biochemistry The City College of New York New York NY 10031 USA; ^2^ CUNY Energy Center The City College of New York New York NY 10031 USA; ^3^ CEMHTI CNRS (UPR 3079) Univ. Orleans 4571 Orléans France; ^4^ Instituto Nacional del Carbon (INCAR) CSIC 33011 Oviedo Spain

**Keywords:** bandgap, nanoporous carbons, photoactivity, photocatalysis, surface chemistry

## Abstract

Even though, owing to the complexity of nanoporous carbons' structure and chemistry, the origin of their photoactivity is not yet fully understood, the recent works addressed here clearly show the ability of these materials to absorb light and convert the photogenerated charge carriers into chemical reactions. In many aspects, nanoporous carbons are similar to graphene; their pores are built of distorted graphene layers and defects that arise from their amorphicity and reactivity. As in graphene, the photoactivity of nanoporous carbons is linked to their semiconducting, optical, and electronic properties, defined by the composition and structural defects in the distorted graphene layers that facilitate the exciton splitting and charge separation, minimizing surface recombination. The tight confinement in the nanopores is critical to avoid surface charge recombination and to obtain high photochemical quantum yields. The results obtained so far, although the field is still in its infancy, leave no doubts on the possibilities of applying photochemistry in the confined space of carbon pores in various strategic disciplines such as degradation of pollutants, solar water splitting, or CO_2_ mitigation. Perhaps the future of photovoltaics and smart‐self‐cleaning or photocorrosion coatings is in exploring the use of nanoporous carbons.

## 1 Introduction

Photochemical reactions are those triggered by the absorption of UV, visible, or infrared radiation.^1^ Most of them proceed through the formation of thermodynamically unstable compounds (e.g., radicals) that overcome large activation barriers during short periods, thus allowing reactivity, which is otherwise inaccessible by conventional methods. A light‐initiated reaction starts with the absorption of light by a molecule or atom, which causes the generation of excited state species that are usually more reactive than those in the ground state.

Many photochemical reactions take place in nature. Examples are photosynthesis, human skin production of vitamin D, ozone formation/dissociation in the atmosphere, or photochemical smog. The concept of photoactivity, so popular in science and technology nowadays, especially in materials science and solid‐state physics, was first introduced at the beginning of 20th century to describe the phenomenon occurring in photovoltaic devices. In the latter, light is absorbed, causing the excitation of an electron, ion, or hole (so‐called charge carriers) to a higher‐energy state. The separation of charges produces an electric potential. An important condition is that the light has sufficient energy to overcome the potential barrier for excitation.

The use of light energy (particularly sunlight) in nonbiological photochemical processes has long been searched, and many ground‐state reactions have been studied (e.g., photocycloadditions, photoisomerizations, and photo‐oxidations). In this regard, photochemical reactions are useful in various fields such as synthetic chemistry (as the excitation of electronic molecular states with high‐energy irradiation may induce chemical bond breaking), cleaning surfaces, solar energy storage and conversion (water splitting for hydrogen generation and carbon dioxide photoconversion into fuels), and environmental remediation (water and air purification).^2^

Many molecules and materials are capable of absorbing light and undergo a chemical or physical change in response to the illumination. The most common photoactive materials are semiconductors having well‐defined absorption features in the UV–vis range. Examples are inorganic oxides or salts such as TiO_2_, ZnO, ZnS, SiC, CdS, CdSe, MoS_2_, WO_3_, Fe_2_O_3_, GaAs, and many others.^3^, ^4^ Other examples are organic semiconductors containing building blocks of π‐bonded molecules or polymers consisting also of, besides carbon and hydrogen atoms, heteroatoms such as nitrogen, sulfur, or oxygen. This group includes phthalocyanine, polytiophenes, or graphitic carbon nitride.^5^

Photochemical reactions in a heterogeneous phase have been mainly associated with wide bandgap semiconductors as photoactive materials. The mechanism of light absorption in these materials is based on the interband electron transitions that occur when photons of adequate energy are adsorbed by a photocatalyst. This generates excitons (neutral, bound electron−hole pairs with properties different from those of excited states due to electrostatic and electron‐exchange interactions with other species), whose fate will determine the efficiency of the photochemical reaction (recombination emitting light or in the form of heat, surface migration, reaction with electron donors or acceptors). In aqueous environments, excitons may react with water and oxygen to generate oxygen‐reactive radical species (hydroxyl and superoxide radicals, singlet oxygen) that undergo further reactions, enhancing the efficiency of the overall photochemical reaction.

Major drawbacks of semiconductors are related to their low efficiency under visible light, the instability upon long irradiation times, and the high recombination rate of photogenerated electron−hole pairs. To overcome these limitations, research efforts have focused mainly on the synthesis of new materials. Explored methods include the incorporation of dopants to modify the optical and electronic band structures of semiconductors and to reduce surface recombination,^6^, ^7^, ^8^, ^9^ surface sensitization with dyes,^10^ synthesis of hybrid photocatalysts, and immobilization on porous supports.^11^

Even though carbon materials are strong light‐absorbing solids, the use of different forms of carbons as supports and additives has been extensively studied as an alternative approach to improve the photoactivity of a variety of semiconductors.^12^, ^13^, ^14^, ^15^ It is generally accepted that the role of a carbon material as an additive to a semiconductor depends on the structural features of the carbon itself. For instance, for carbons with a high electron mobility (e.g., carbon nanotubes and graphene), the enhanced photoactivity of semiconductor/carbon composites is mainly attributed to strong interfacial electronic effects between the carbon material and the semiconductor that favor the separation of the photogenerated charge carriers through delocalization in the π‐electron density of the carbon matrix.^13^, ^16^, ^17^ In the case of porous carbons, the enhanced mass transfer in the pores and the nanoconfinement of the adsorbed species also play important roles.

Discovery of carbon nanotubes and graphene soon directed the attention of scientists to their optoelectronic properties.^18^, ^19^, ^20^ Monolayer or double‐layer graphenes are considered as gapless semiconductors. In 2009, Luo et al. reported the self‐photoactivity of carbon nanotubes under visible light, attributing the behavior to the presence of structural defects and vacancies.^21^ Later, other authors reported the ability of aqueous suspensions of carbon nanotubes to generate reactive oxygen species (ROS) including singlet oxygen, superoxide anion, and hydroxyl radicals upon illumination with light within the solar spectrum.^22^, ^23^, ^24^

The unique optical features of graphene boosted the study of the photoactivity of graphene and its composites with semiconductors. The incorporation of graphene layers to semiconductors has been reported to significantly increase the photoactivity of the latter. This effect is mainly explained by the graphene phase providing electrical conductivity, separating charge carrier, and delaying their recombination. However, the beneficial effect of the use of graphene for photocatalytic purposes is still quite controversial, and has been recently argued that its enhancing role in the photocatalytic activity of TiO_2_/graphene composites is in essence the same as that of any other carbon material.^25^ Here, it is important to mention that in fact the addition of an amorphous carbon phase to semiconductor for increasing the photoactivity was initiated well before the graphene discovery.^14^, ^26^, ^27^

Interestingly, despite the structural and chemical similarities between amorphous carbons and graphene, there has been a dearth in exploring the self‐photochemical activity of these forms of carbon. In this regard, recent works by Velasco et al. have reported the photochemical activity of aqueous suspension of nanoporous carbons (metal free and semiconductor free) as well as their ability to generate ROS when exposed to UV–vis light.^28^, ^29^, ^30^, ^31^ The authors highlighted the important effect of nanoconfinement in the pores of the carbon material, reporting high photochemical quantum yields for the degradation of aromatic compounds in the confined pores of the carbons.

Even though an amorphous carbon phase certainly differs from that of graphene, it might have certain advantage when the photoactivity is concerned. Their heterogeneity (disorders/defects) and the presence of sp^2^ and sp^3^ configurations directed the attention of scientists to study the origin of the bandgap with intended applications in photovoltaics as amorphous semiconductors. This important optical property was linked to the size of sp^2^ units, π–π∗ transitions, and disorder caused by sp^3^ configurations.^32^, ^33^, ^34^, ^35^, ^36^, ^37^, ^38^

Another advantage of amorphous carbon is that it can exist in a 3D structure reaching surface areas over 1000 m^2^ g^−1^.^39^ Even though the high volume of the fraction of nanometric pores provides such a high surface area, nanoporous carbon is known for having a hierarchical pore structure. Recent studies have shown that their pore walls are built of thin distorted graphene layers which, besides sp^2^ domains,^40^, ^41^ are also rich in defects/heteroatoms and sp^3^ configurations. Even though their electrical conductivity is lower than that of graphene or carbon nanotubes,^39^, ^42^, ^43^ it is still much advantageous than that of inorganic semiconductors. Thus, for photocatalysis, such features are considered favorable for promoting the electron/hole transfers or radical reactions on the substrates immobilized in the pore space of strong adsorption potentials. That adsorption potential can additionally contribute to bond splitting.

**Figure 1:**
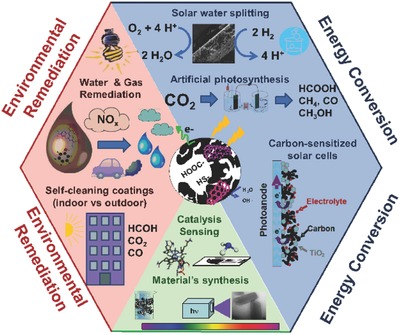
Current and forecasted application fields for the photochemical activity of nanoporous carbons.

Although the application of nanoporous carbons themselves as photocatalysts or photosensitizers is still in its infancy, there is a strong indication that visible light activates their surface centers, generates charge carriers, which either directly or indirectly (via radical formation) not only increase the efficiency of chemical reactions but also contribute to energy applications such as charge storage in a supercapacitor. Thus, the objective of this review paper is to summarize the recent findings with the emphasis on the role of the carbon surface features in the photoactive/photosensitizing behavior and to indicate the advantage of their texture for these applications. We limit the scope of this review, especially concerning the photochemical applications, to amorphous nanoporous carbons. We also point out specific directions of surface modifications, which are beneficial for visible light harvesting and utilization of this group of materials. **Figure** **1** presents our vision on current and forecasted applications of photoactive carbons inspired by the recent works of various groups on this topic; the reader will find the detailed bases for this listing throughout this paper. Even though the porous/nanoporous carbons are old materials of an ancient origin and application (chars), we believe that their “new life” is still in front of them, and being of low costs and environmentally benign are certainly pluses from economic and environmental conservation viewpoints.

## 2 Origin of Photoactivity of Nanoporous Carbons

### 2.1 Optical and Electronic Bandgap

Amorphous carbons can be considered as semiconducting materials, and although some theoretical attempts have been published to explain their optical properties, there is still some ambiguity related to their electronic bandgap. This is related to the lack of well‐defined conduction band (CB) and valence band (VB) in this kind of materials and to the complexity of their structure and chemical buildup. Furthermore, the solid‐state physics reports rather address the optical bandgap and not the electronic one. Despite a relationship between both concepts, it is important to have in mind the difference between them.

From the concept of a band structure in the physics of solids, an energy gap or bandgap is a range where no electron states can exist. This generally represents the energy required to move an electron in a bound state in the VB of the solid to the CB. In photochemistry, the energy input is provided by the absorption of photons. Indeed, the absorption of a photon can create an exciton (bound electron–hole pair), but the energy might not be high enough to split the electrically bounded electron and hole allowing them to become free charge carriers in the CB. Thus, while the optical bandgap is the threshold for photons to be absorbed, the electronic bandgap is the threshold for separating the photogenerated bound excitons. The evaluation of one or the other depends on the application being targeted; the optical bandgap measurement is important for solar cells applications, and the electronic bandgap for light‐emitting diodes and laser diodes. In most inorganic semiconductors, both bandgaps are essentially similar due to the low binding energy. In the case of carbon materials and organic semiconductors, differences may be significant; the energy of the optical bandgap is usually lower than that of the electronic bandgap. It is owing to the strong attraction of electron–hole pairs (high exciton binding energies).

Even though numerous works exist on optical properties of graphene or carbon nanotubes,^19^, ^20^ we limit the discussion here to amorphous carbon films and nanoporous carbons. The former, although they might differ from the latter ones in the amount of hydrogen, show some similarities to nanoporous amorphous carbons, which are the main materials addressed in the review.

In the late 70s and at the beginning of the 80s of the last century, an increasing need for cost‐efficient semiconducting/photovoltaic devices directed the attention of scientists to amorphous semiconductors, and amorphous carbon was considered to be in this category of materials. One of the first efforts to investigate conductivity and optical properties of carbon film was undertaken by Anderson.^44^ His amorphous carbon films had an optical gap of 2 eV and it depended on the synthesis temperature. The latter was associated with changes in regions of graphitically and tetrahedrally bonded carbon atoms.

Following the efforts of Anderson, Meyerson and Smith^34^ studied the optical properties of hydrogenated amorphous carbon films. The films were transparent and obtained at relatively low temperatures between 75 and 375 °C. They were rich in hydrogen, and an increase in the synthesis temperature (*T* > 250 °C) resulted in an increase in the contribution of sp^2^ configuration, in the loss of hydrogen, and thus in the ten order of magnitude increase in the conductivity (from 10^−16^ to 10^−6^ Ω cm^‐1^ for the films synthesized at 75 and 350 °C, respectively). The authors proposed that the electrical conductivity of these films involved thermal excitation into a broad range of localized energy states at one of the band edges where conduction took place via thermally activated hopping. Their results also suggested the existence of various energy states at the band edges and the dependence of the conductivity, and the positions of the valence and the conduction band on the synthesis temperature. The latter affected the coordination state of the carbon atoms (increase in the sp^2^ level). The optical energy gap for the films studied by Meyerson and Smith was between 2.2 and 0.9 eV, and decreased with an increase in the synthesis temperature. Similar optical gaps were also measured by Dischler et al.^32^ and by Fink et al.^33^ on their amorphous carbon films. Kaplan et al.^45^ reported the dependence of the optical bandgap in amorphous carbon on an sp^2^/sp^3^ ratio: while for sp^2^/sp^3^ = 0.16, it was 4.1 eV, for sp^2^/sp^3^ = 1.63 it was 1.2 eV.

The origin of the bandgap in amorphous carbons was first discussed by O'Reilly et al.^46^ They analyzed the density of states (DOS) for diamond (5.5 eV), graphite (0 eV), and randomly built network of carbons with 100%, 86%, and 52% of sp^2^ sites (graphitic layers with five‐ and seven‐membered rings) and indicated that the bandgap is created by the disorder of π states. The authors proposed that a bandgap exists in the structures consisting of partially bounded graphic regions, and that it is inversely proportional to the size of these graphitic regions. A bond alteration was also indicated as contributing to the bandgap formation. Their calculation showed that those disorder mechanisms were capable of creating a bandgap of 0.7 eV for a system with a small proportion of sp^3^ sites.

Robertson and O'Reilly^35^ further continued their study on the origin of the bandgap in amorphous carbon by performing detailed calculation of the DOS of model systems consisting of various contributions of sp^2^ and sp^3^ configurations. Following the work of Wada et al.,^47^ they based their calculations on an assumption that the structure of amorphous carbon consists of sp^3^ states in the form of disordered graphitic islands of 1.5–2 nm in diameters with 34–60 rings, and these islands are connected by sp^3^ sites. Nevertheless, the presence of the latter was not sufficient to produce an optical gap. The π states were found as very important, and the size of the optical gap was inversely proportional to the sizes of sp^2^ clusters. The calculations of Breads and Street^48^ were consistent with those of Robertson and O'Reilly.^35^

**Figure 2:**
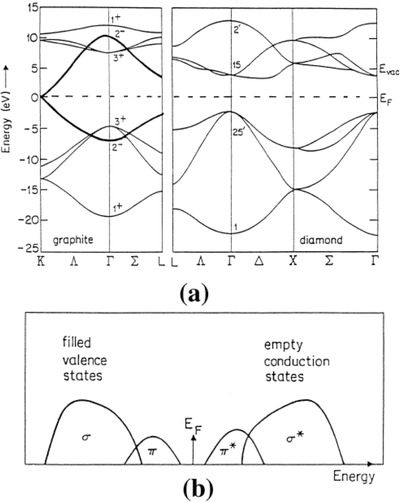
a) schematic electronic energy band structures of graphite and diamond (the graphite π states are indicated by heavy lines). b) a‐C:H (carbon film) schematic electronic density of states deducted from the cluster model. Reproduced with permission.^38^ Copyright 2002, Elsevier.

The electronic density of states in hydrogenated amorphous carbon films was also studied by Theye and Paret^38^ (**Figure** **2**). Their calculations indicated that the type of clustering of the sp^2^ carbon atoms (microstructure) has more influence on the electronic density of states and the magnitude of the optical bandgap than the proportion of the carbon atoms in this configuration. That microstructure has little effect on σ state density. The semiconducting properties of the carbon samples were linked to the limitation in the size of sp^2^ clusters. The authors also indicated that σ and π states in amorphous carbons form independent sub‐bands in both the valence and the conduction band. Moreover, the existence of states with an intermediate hybridization and/or transition between π and σ states was also suggested.

**Figure 3:**
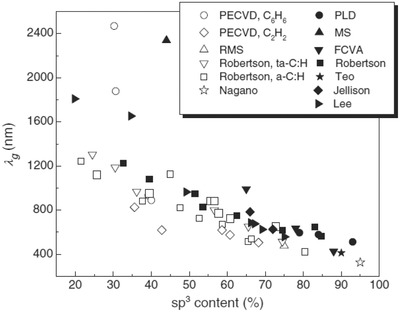
The wavelength of light corresponding to the fundamental gap for various a‐C (solid symbols) and a‐C:H (hollow symbols) films grown by various techniques. Reproduced with permission.^49^ Copyright 2011, Elsevier.

A recent review on the optical properties of amorphous carbons, and their application and perspectives in photonics by Patsalas,^49^ presents a comprehensive summary of the solid‐state physics approaches (**Figure** **3**). By collecting own^50^ and other authors' data, the study shows that the optical gap of amorphous carbons increases with the sp^3^ content (or density). The authors also indicated that merely introducing sp^3^ sites does not automatically create a gap; they observed a correlation between the optical properties of amorphous carbon films and some other structural features (e.g., distribution of sp^2^ states, dangling bonds, and defects), inferring that all these features should be taken into consideration for the preparation of photonic devices based on amorphous carbons. Similar conclusions were further corroborated by other authors^35^, ^36^, ^37^, ^46^, ^49^, ^50^, ^51^ on amorphous carbons and graphites.

The optical properties of hydrogen‐free amorphous carbon films with incorporated nitrogen were also studied by Miyajima et al.^51^ They applied various experimental techniques (including electron energy loss (EELS), scanning tunneling (STS), photoelectron (X‐ray photoelectron spectroscopy (XPS) and ultraviolet photoelectron spectroscopy (UPS)), photothermal deflection (PDS), optical transmittance spectroscopies) to measure the DOS, with differences among them. They found that the incorporation of nitrogen increases the width of the π* band and the slope of the conduction band. These changes were associated with the existence C—N bonds and were consistent with a decrease in the optical gap. Interestingly, the presence of nitrogen increased the Tauc gap by about 0.2 eV, which was linked to defect tail states. The electrical bandgap measured by scanning tunneling microscopy was about 2 eV and much higher than the optical bandgap. Long band tail states contributed to it. A band tail hoping at low fields was suggested as a conduction mechanism, and sp^2^ clusters were indicated as hopping centers. The films without or with nitrogen were found to be a slightly p‐type materials.

Adhikari et al. investigated the optical bandgap of nitrogenated amorphous carbon films.^52^ They found that by nitrogen doping the *E*_g_ of the films decreased from 4.1 to 2 eV and from 2.4 to 1.6 eV, depending on the carbon precursor. Thermal annealing further decreased the bandgap to 0.95 eV. They also noticed a decrease in *E*_g_ with an increase in the nitrogen content, and it was explained by the formation of sp^2^ hybrids where π states are weakly boned and thus lie closer to Fermi level than the σ states.

**Figure 4:**
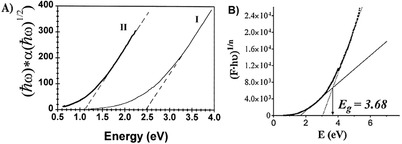
a) [*ℏω*.α (*ℏω*)]^1/2^ versus photon energy for a‐C:H films deposited at I) 30 V and II) 600 V; lines represent fitting to Tauc equation. Reproduced with permission.^38^ Copyright 2002, Elsevier. b) Bandgap energy calculation for an activated carbon using spectrophotometric measurements. Reproduced with permission.^53^ Copyright 2013, Elsevier.

Velo‐Gala et al. have reported values of optical bandgap for a series of activated carbons with varied chemical compositions using UV–vis–NIR diffuse reflectance spectroscopy^53^ (**Figure** **4**). The authors found values ranging from 3.1 to 3.5 eV; they also reported a strong effect of the extent of surface carbon oxidation, with lower bandgap values in carbons with high surface oxygen contents. It should be noted that the values of optical bandgap reported by Velo‐Gala et al. for activated carbons are significantly higher than those reported by Miyajima et al.^51^ or Theye and Paret^38^ for other types of amorphous carbons (Figures 3 and 4). This could be attributed to the limitations of the spectrophotometric method and the validity of the mathematical approach used to fit the Tauc equation. Although UV–vis–NIR diffuse reflectance spectroscopy based on the application of the Kubelka–Munk theory is one of the most popular method for evaluating the optical bandgap of semiconductors, there is no unanimous consensus regarding the best mathematical approach of this data interpretation.^54^, ^55^ A recent comparative study on TiO_2_ has pointed out the choice of an appropriate graphical method as the main challenge to obtain reliable data through the use of the Tauc equation.^55^ Another controversial issue is the incertitude concerning the value assigned to the exponent in the Tauc equation that takes into account the type of transitions in the material (direct, indirect, allowed, or forbidden). For amorphous carbons this is most outstanding given the dearth in the understanding of the origin of these transitions. Theye and Paret^38^ also discussed the applicability of this method to hydrogenated amorphous carbon films, inferring that the Tauc gap must be considered as a phenomenological parameter to be used to compare different samples.

**Figure 5:**
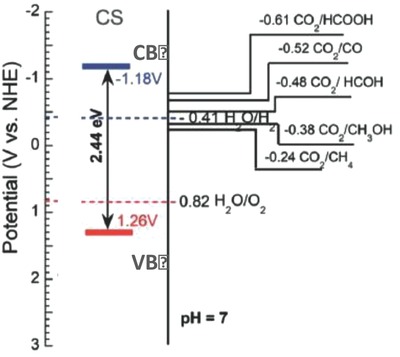
Bandgap and position of the VB and CB for nanoporous carbon CS. Adapted with permission.^59^ Copyright 2017, The Royal Society of Chemistry.

Another method recently used to estimate not only the position of the energy bandgap but also a type of charge carries in nanoporous carbons is based on impedance spectroscopy measurements and the Mott–Schottky approach.^56^ The studies on S‐, N‐, and O‐containing nanoporous carbons placed the bandgap between 1.4 and 2.9 eV.^57^, ^58^, ^59^ The positions of CB and VB were indicated depending on the microstructure and chemistry of the carbon materials (**Figure** **5**). While holes were found as predominant charge carriers in nonmodified carbons (p‐type), certain modifications, for instance, an introduction of electron donating moiety as —NO_2_ groups, were found as converting carbon to n‐type semiconductors.^60^ The authors also observed the coexistence of both p‐ and n‐type charge carriers in functionalized nanoporous carbons. The transition from n‐type to p‐type materials upon functionalization has been reported for carbon‐based materials, including graphene nanoribbons.^61^, ^62^ Gomis‐Berenguer described a similar behavior on nanoporous carbons with a low functionalization level, reporting frequency‐dependent impedance measurements for some carbons.^63^ Furthermore, the Mott–Schottky approach showed the response of either p‐ or n‐type for some carbon materials, depending on the frequency range analyzed. It should be mentioned that the nature of this behavior remains unclear, since the validity of the Mott–Schottky approach and the optimal frequency region for nanoporous carbons—with large double‐layer capacitances—is under debate.

### 2.2 Nature and Reactivity of the Charge Carriers in Nanoporous Carbons

Besides an energy bandgap, another important feature that may control the application of a material with photochemical activity is the nature and strength of the electron–hole binding, as well as the fate of the excitons generated after irradiation. As mentioned above, the optical energy gap controls the energy of the photons, while the electronic bandgap controls the separation of the bound electron–hole pairs, thus their ability to allow further reactions. Such an ability to be separated is controlled by the exciton binding energy (i.e., electron–hole interactions) that depends on the properties of the material.

#### 2.2.1 Nature of Excitons

To further comprehend and discuss the photoactivity of nanoporous carbons, fundamentals on the nature of excitons are discussed in this section. Mainly two types of excitons (Wannier–Mott and Frenkel) can be differentiated.^64^ In materials with high dielectric constants such as most inorganic semiconductors, Wannier–Mott excitons (also known as free, large, or weakly bound) are mainly formed. These are delocalized states with a low binding energy (≈0.01 eV) that can move through the crystal of the solid (radius ≈ 10 nm), due to reduced Coulombic interactions between the electrons and the holes. In Wannier–Mott excitons, the bound electron–hole pair is not localized at a crystal position; thus, it can easily diffuse across the lattice of a semiconductor. In contrast, in materials with small dielectric constant (e.g., insulators and molecular crystals), Frenkel excitons (tightly bound) are formed. These are localized states due to the strong Coulombic interactions between the electrons and the holes (binding energy ≈0.1–1 eV). They can diffuse by hopping from one atom to another (radius ≈1 nm). Frenkel excitons are typically found in alkali halide crystals and in organic molecular crystals composed of aromatic molecules, such as anthracene. Charge transfer excitons and molecular excitons have also been reported for ionic crystals, organic materials, and fullerenes. The first one is an intermediate case between Frenkel and Wannier excitons, occurred when the electron and the hole occupy adjacent molecules. The latter occurs when the excitons is entirely located on the same molecule, and it has been reported for fullerenes and their blends with carbon nanotubes (CNTs).^65^

Since the first observation of the C1s‐core exciton of diamond,^66^ much theoretical as well as experimental work has been devoted to the understanding the electron–hole interactions of carbon materials, particularly to unravel whether they should be considered as Wannier‐ or Frenkel‐type excitons. The answer to this question is not trivial since different types of excitons may also exist in the same material. For instance, in single‐wall carbon nanotubes' excitons may have both Wannier–Mott and Frenkel characters depending on the chirality and the different screening of Coulomb interactions in 1D systems.^67^ The dielectric function of the nanotubes is large enough to allow for the spatial extent of the wavefunction to extend over a few to several nanometers along the tube axis, while poor screening in the vacuum or dielectric environment outside of the nanotube allows for large (0.4–1.0 eV) binding energies.^65^ On the other hand, Gutierrez and Lopez described Frenkel‐type excitons in amorphous carbon films, accounting for the core exciton in sp^3^‐hybridized carbon atoms.^68^ The authors also reported that disorder may induce the localization of the excited state, shifting the binding energy of the excitons toward higher values.

Based on the low dielectric constant of nanoporous carbons and their low functionalization level—compared to n‐type semiconductors—medium‐to‐low range excitons (Frenkel‐like created in the π–π* and σ–π electronic transitions involving zigzag, carbine‐like sites and other intermediate states) have been postulated as the dominant electronic transitions upon illumination in the UV–vis range.^63^, ^69^ In functionalized carbons, Frenkel‐like excitons would be less stable due to the presence of structural defects and changes in the sp^2^/sp^3^ hybridization state of carbon atoms provoked upon functionalization.^63^, ^69^, ^70^ Besides, charge transfer excitons may also be formed by localized states involving O‐, S‐, and C‐atoms; electron transfer is facilitated since the energy difference between the electronic levels is lower than that in pure carbon units.^71^

The pore size and the confinement state of electron donors/acceptors also play an important role in the ability of the carbon material to convert light into chemical reactions. Weak molecule/pore wall interactions in large nanopores decrease the probability of splitting of the photogenerated exciton through fast charge transfer reactions with electron donors and/or holes scavengers.^63^, ^69^, ^72^

#### 2.2.2 Charge Carrier Stabilization Routes

After the absorption of light and formation of excitons, their lifetime and ability to recombine and/or split to react with electron/donors are critical factors to boost an efficient conversion of light into chemical reactions. The stabilization of the excitons can occur via several processes such as surface recombination, radiative and nonradiative relaxation (vibration, internal conversion, and heat dissipation) that either do not involve chemical reactions or provoke a chemical transformation in the material itself. Another possibility is a photosensitization process, where the stabilization of the excitons occurs through a reaction with different molecules.

Depending on the mechanism followed, two types of photosensitization reactions can occur. In type I reaction, the excited sensitizer reacts directly with a substrate, producing radical species in both the sensitizer and the substrate.^1^, ^73^ In the presence of oxygen and water, both radicals can further react to produce further ROS (e.g., superoxide and hydroxyl radicals) or the oxidation of the photosensitizer, eventually producing its deactivation by a photobleaching mechanism. In type II reaction, the excited sensitizer directly reacts with molecular oxygen to produce singlet oxygen, which, in a second step, reacts with the target substrate (the photosensitizer is not consumed during the photosensitization reaction).

The stabilization of the electron–hole pairs through chemical reactions with other molecules, following a photosensitization mechanism, has been much investigated for carbon nanotubes.^74^, ^75^, ^76^ Aqueous colloidal dispersions of bare and functionalized CNTs have been reported to generate ROS including singlet oxygen, superoxide anion, and hydroxyl radicals in light within the solar spectrum under oxic conditions. Purified CNTs were found to be generally more photoactive, producing singlet oxygen and superoxide, while little or no photoactivity was observed for raw CNTs.^22^, ^23^, ^24^ CNTs can also act as scavengers of hydroxyl or superoxide radicals,^74^, ^75^, ^76^, ^77^ or undergo photochemical transformations (e.g., decarbonilation) as in the case of oxidized multiwalled CNTs.^24^

**Figure 6:**
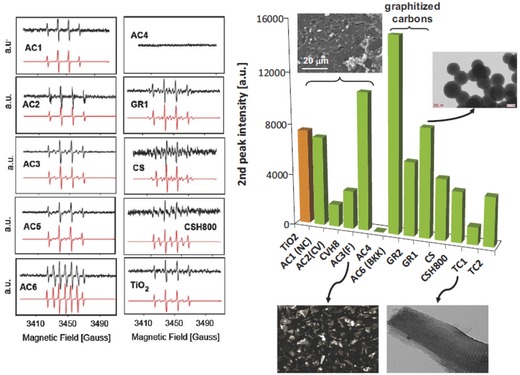
Left: Experimental (top, black) and simulated (bottom, red) ESR spectra of selected nanoporous carbons and commercial titania powders (P25, Evonik). Reproduced with permission.^31^ Copyright 2017, Elsevier. Right: Quantification of the radical species detected from the ESR signals corresponding to DMPO–OH adducts (second peak intensity in the 1:2:2:1 patterns) for the nanoporous carbons (selected transion electron microscopy (TEM) and scanning electron microscopy (SEM) images of the carbons are also shown for clarity). Reproduced with permission.^79^ Copyright, 2017, Elsevier.

Velasco et al. studied the possibility of the formation of ROS upon illumination of a variety of nanoporous carbons (i.e., microporous carbons, carbon nanotubes, expanded graphite, porous carbon hydrochars, ordered micro/mesoporous carbons) under UV–vis light using electron spin resonance spectroscopy (ESR) and nitrones as chemical trapping agents (e.g., 5,5‐dimethyl 5,5‐dimethyl‐1‐pyrroline N‐oxide (DMPO)) (**Figure** **6**).^29^, ^30^, ^31^ Most carbons showed similar ESR patterns, with the characteristic quartet peak profile with a 1:2:2:1 pattern of intensities corresponding to DMPO–OH adducts.^78^, ^79^ Some other patterns associated with HDMPO–OH, DMPO–R (carbon‐centered radical), and DMPO–OOH adducts were also identified for some carbons, while no signals were detected for some other carbons (Figure 6). A correlation was found between the formation of ROS and the acidic/basic nature of the nanoporous carbons. As an example, the amount of ROS correlated well with the CO‐evolving groups of a basic nature, while strong acidic groups (CO_2_ evolving) seemed to inhibit the formation of radicals. Most interestingly, some of the studied nanoporous carbons presented higher relative abundance of ROS than inorganic semiconductors (e.g., TiO_2_) under similar experimental conditions.

Velo‐Gala et al. measured the photogeneration of ROS on activated carbons with varied chemical composition upon UV and solar irradiation.^80^ The authors found that the amount of ROS depended on the surface chemistry of the activated carbons and concluded that activated carbons of a higher photoactivity are those having large amounts of chemisorbed oxygen on the surface. Furthermore, the formation of hydroxyl radicals over superoxide anion was favored in activated carbons of low optical bandgap values.

The detection of spin‐trapping adducts confirmed the formation of hydroxyl and/or superoxide radicals upon the irradiation of the nanoporous carbons in an aqueous medium, upon the stabilization of the photogenerated charge carriers. The stabilization of the holes through the oxidation of water would form hydroxyl radicals, while the electrons participate in the multielectron reduction of molecular oxygen to produce superoxide radicals and hydrogen peroxide, which may be further reduced to hydroxyl radical or water. However, it should be taken into consideration that ESR spectroscopy just provides a diagnostic indication of the formation of free radicals and the likely predominance of a radical‐mediated process, and not of the photoactivity of the carbon material. In this regard, some functionalized carbons showing a low concentration of ROS displayed the highest photocatalytic activity toward phenol photo‐oxidation.^31^ A similar behavior has been reported for some titanium oxide powders.^81^

The dynamics and lifetime of the transient species generated upon irradiation of a given material are key parameters in determining their implications. Time‐resolved laser flash photolysis is a powerful technique for the investigation of transient species, and has been largely employed to investigate the electron donor or electron acceptor in intermolecular photochemical conversions involving semiconductors,^82^, ^83^ as well as C60 and CNTs.^84^, ^85^ Wu et al. reported the transient absorption spectra of CNTs in pure water, demonstrating that CNTs could be photoionized and trap hydrated electrons upon high‐energy irradiation.^85^ Under UV irradiation, CNTs could promote the generation of ROS such as singlet oxygen and hydroxyl radicals. In the presence of H_2_O_2_, the hydroxyl scavenging effect predominated in the aqueous medium. As a result, the presence of CNTs suppressed the photodegradation of a dye, due to the scavenging effect. Vizuete et al. also reported the role of functionalization of carbon nanotubes on the lifetime of photogenerated transient species. Shorter‐lived transients were observed for pentyl‐esterified functionalized double‐wall CNTs with respect to single‐walled CNTs, due to charge recombination processes in the inner (nonfunctionalized) graphene wall of the former.^86^

For nanoporous carbons, Gomis‐Berenguer et al. evaluated the lifetime of the transient species formed upon irradiation of aqueous suspensions of nanoporous carbons using time‐resolved absorption spectroscopy.^87^ Laser flash photolysis of deoxygenated aqueous suspension of nanoporous carbons after a 266 nm pulse led to the formation of a detectable transient after 29 ns, of broadbands with maxima at 470 and 550 nm, which can be attributed to trapped solvated holes and electrons.^83^, ^88^ The strong absorption changes that appeared promptly 29 ns after the laser pulse decayed rapidly within ≈200 ns, confirming the predominance of short‐lived (Frenkel‐type) excitons. Spectra recorded for aqueous suspensions of TiO_2_ powders pointed out the occurrence of both long‐lived (Wannier) and short‐lived (Frenkel) excitons.^89^ Furthermore, in the presence of an electron scavenger (e.g., methyl viologen) bleaching of the signal at 470 nm was observed, along with the increase in the signal at 600 nm, corresponding to the reduced form of the scavenger, thus confirming that the electron scavenger concentration is reduced upon reaction with the photogenerated electrons.^90^, ^91^ The initial fast decay of the transient absorption obeys a first‐order kinetics, and the exponential data fit of the absorption/time signal revealed a decay constant between 35 and 50 ns (depending on the wavelength). The values were smaller than those reported for inorganic semiconductors.

#### 2.2.3 Proposed Mechanisms of Electronic Transitions in Nanoporous Carbons

**Figure 7:**
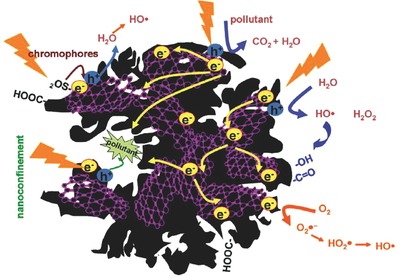
Schematic representation of the mechanism proposed for the exciton's formation and fate upon irradiation of nanoporous carbons. Reproduced with permission.^79^ Copyright 2017, Elsevier.

Based on the experimental evidences of the photochemical activity of nanoporous carbons of varied physicochemical properties (e.g., texture, composition, and structure) under various illumination conditions, several hypotheses have been proposed to account for the electronic transitions occurring upon irradiation of nanoporous carbons (**Figure** **7**). The absorption features of amorphous nanoporous carbons have been reported to depend on the sp^2^/sp^3^ hybridization ratio of the carbon atoms, the electronic transitions involving sp^2^ carbon clusters,^35^, ^36^, ^37^ and/or the activation of chromophoric groups on the carbon surface.^70^, ^92^ The size and ordering of the sp^2^ clusters, their population in the carbon matrix, and the creation of distortions of π* states have been suggested to have a strong effect in the optical characteristics of the carbons. Upon irradiation of the carbons, these electronic transitions generate excitons (holes or electrons) that can be delocalized through the sp^2^ conjugated domains of basal planes of the nanopore walls, avoiding or delaying recombination phenomena. This favors their participation in other electronic transitions with acceptors/donors such as water, oxygen, surface groups, or other molecules, either involving a direct hole/electron scavenging or a radical‐mediated mechanism.

Regarding the nature of the photogenerated charge carriers, in the UV range, medium‐to‐low range excitons (Frenkel‐like created in the π–π* and σ–π electronic transitions involving zigzag, carbine‐like sites, and other intermediate states) are expected to be dominant in nanoporous carbons of a low functionalization level.^69^, ^93^ This is consistent with the behavior reported for other carbons nanostructures such as carbon blacks, graphene, or fullerenes of higher polarity and electron mobility, based on experimental observations and molecular simulations.^94^, ^95^, ^96^

In functionalized carbons, charge transfer excitons formed by localized states involving O‐, S‐, N‐, and C‐atoms may also be formed. Additionally, N‐, S‐, and O‐containing groups can act as chromophores that may be activated under sunlight irradiation, photogenerating vacancies (holes) able to accept electrons from oxygen in water molecule that may participate further in photochemical reactions, as proposed for various oxidized forms of sulfur (e.g., thioesters and sulfones) and O‐containing groups.^95^, ^97^ The nature of the surface moieties of the nanoporous carbons is also important; for instance, bulky oxidized sulfur moieties (i.e., thioesters and sulfones) located at the entrance of the wide micropores seem to be more photoactive under visible light than thiols and/or sulfide groups.^63^

Hence, the varied chemical environments of the surface functionalities present on nanoporous carbons, as well as the different distribution of pore sizes, can explain differences in their photochemical response. On the one hand, the light conversion in nanoporous carbons is boosted in the tight nanopore space, pointing out the outstanding role of the confinement state of the adsorbed molecules. Weak molecule/pore wall interactions in large nanopores decrease the probability of splitting of the photogenerated exciton through fast charge transfer reactions with electron donors and/or hole scavengers (i.e., oxygen, adsorbed phenol, and water molecules to form radicals).^30^, ^31^, ^80^ On the other hand, low photoactivity of functionalized nanoporous carbons has been associated with several factors such as the creation of structural defects, changes in the sp^2^/sp^3^ hybridization state of carbon atoms during the functionalization treatment, or the lower stabilization of the photogenerated carriers due to the withdrawal effect of surface groups (such as carboxylic acid and anhydrides) on the π‐electron density of the conjugated sp^2^ network.

### 2.3 Insights on Photocurrent Extent in Nanoporous Carbons

**Figure 8:**
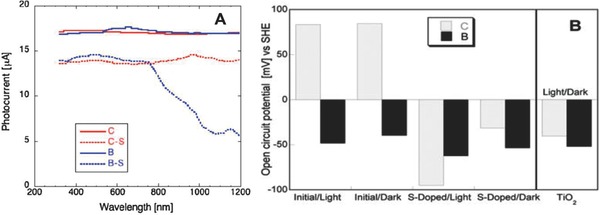
a) Photocurrent generated by the carbons studied. b) Comparison of the open‐circuit potential: C and B represent two different types of nanoporous carbons. Reproduced with permission.^92^ Copyright 2012, Elsevier.

The ability of sulfur groups incorporated into nanoporous carbons to alter the energy gap^71^ triggered a direct investigation of the photoactivity of sulfur‐doped carbons. They had the surface areas over 1000 m^2^ g^−1^ with micropore volumes of ≈0.5 cm^3^ g^−1^, and contents of sulfur of ≈2.1 wt% (S‐doped carbon B, BAX‐1500 from Mead Westvaco treated with H_2_S at 800 °C) and 3.1 wt% (S‐doped carbon C, home‐made polymer‐derived carbon).^92^ The photocurrent generated versus the wavelength, PC(λ), for different nanoporous carbon films tested is presented in **Figure** **8**. It was measured in a broad range of irradiation energy and the carbons' activity, however smaller than that for commercial silicone‐based photovoltaic cell,^98^, ^99^ also covered a very broad range of photons energy.

The open‐circuit potential (OCP) values measured under light and in the dark in 0.5 m Na_2_SO_4_ are compared in Figure 8. Apparently light exposure resulted in a fall of the OCP in the negative range, suggesting the capability of S‐doped carbons for oxidation reactions when exposed to visible light. This behavior is typically observed for n‐type semiconductors, although the potential drop for the latter is usually sharp and fast, followed by a steady state.^100^ That photoactivity was clearly demonstrated in the efficiency of methanol blue oxidation under visible light, which was two times higher than that on titania.^92^

Gomis‐Berenguer investigated the photocurrent response of nanoporous carbon electrodes of varied origin by chronoamperometry and open‐circuit potential measurements under dark and light conditions^63^ in a neutral aqueous electrolyte. When the electrodes were illuminated, the open‐circuit potential progressively dropped toward more negative potential values, evidencing the separation of photogenerated charge carriers. The potential drop was slow—compared to the photo‐electrochemical response observed in n‐type semiconductors—which was attributed to the porosity of the carbon electrodes. When the light was switched off, the electrodes progressively recovered their rest potential at dark conditions, also with a rather slow kinetics. The transient photocurrent response under on/off illumination conditions at various bias potentials also showed a photocurrent of various magnitudes for the nanoporous carbon electrodes. The shape of the transient responses showed anodic overshoots and cathodic undershoots in the electrodes. This behavior, frequently reported for semiconductor materials, is an indicator of insufficient mass transport, poorly efficient reactions, and photocorrosion phenomena.^100^, ^101^ The cathodic photocurrent was attributed to the reduction of O_2_ and/or HO• radicals in the pores of the carbon electrodes at negative bias potentials, while the anodic photocurrent corresponds to water oxidation.

Interestingly, one of the carbons on which a photocurrent response was detected showed no photocatalytic activity for the oxidation of phenol in solution under UV–vis light,^102^ pointing out the importance of the target reaction. This particular nanoporous carbon shows photo‐electrocatalytic activity toward the reduction of oxygen and the oxidation of water, but not for the photo‐oxidation of phenol in solution (without a bias potential).

**Figure 9:**
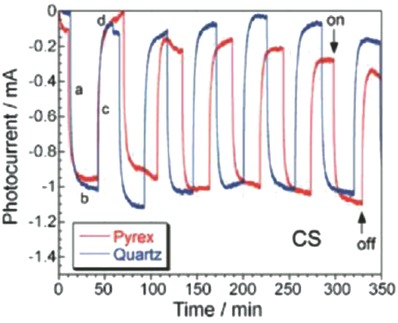
Chronoamperometric response of S‐doped carbon upon on/off illumination at 1 V. Adapted with permission.^58^ Copyright 2016, Wiley‐VCH.

Seredych and Bandosz explored a direct photocurrent generation in S‐doped nanoporous carbons.^58^, ^103^ That carbon was chosen based on its earlier detected photoactivity.^104^ Exposure of the S‐doped carbon to visible light (through Pyrex filter) at 1 V resulted in the generation of a photocurrent response of 0.93 mA (**Figure** **9**). Switching to dark and subsequently re‐exposing to visible light decreased the generated current to 0.81 mA, indicating the instability of the carbon in the first run. The effects of solar and visible light were very similar. A decrease in a photocurrent upon irradiation suggested that the photons move electrons from the conduction band to higher energy electronic localized states, which caused a decrease in carrier density and increased the number of holes as charge carriers. Fitting of the curve to two exponential decay components related to two relaxation processes indicated that the first process representing a recombination time of holes and electrons formed as a result of photoactivity was fast with a relaxation time (*t*) of about 18 s and the second one related to an excitation of electrons to higher energetically metastable states (heteroatom (sulfur and oxygen)‐containing surface groups) was much longer *t* = 224 s. Exposing the sample to UV shortened both relaxations to 8 and 114 s, respectively, as photons with higher energy are considered as re‐establishing the equilibrium easier. Upon switching off the light, the values recovered owing to the return of electrons from traps to conducting band.

Similar results were obtained when S‐doped nanoporous carbon was enriched with nitrogen by the carbonization of its precursor mixed with g‐C_3_N_4_.^52^ In this case, graphitic carbon nitride was used as the N‐dopant of the carbon phase. Even though the carbon itself showed the higher photocurrent generation in solar light than in visible light, an incorporation of nitrogen species from the g‐C_3_N_4_ component diminished these differences. An enrichment in surface chemistry and the incorporation of oxygen was found as widening the bandgap which was reported to be between 1.48 and 2.11 eV.

## 3 Applications of the Photochemical Activity of Nanoporous Carbons

After the pioneering works in 1970s reporting the photocatalytic activity of ZnO and TiO_2_ to decompose cyanides^105^, ^106^ and to promote the photo‐electrochemical breakdown of water,^107^ the interest in the potential applications of photoactive materials has largely increased, and it has become one of the most popular topics at the forefront of technology in various disciplines; particularly in the fields of solar light conversion for environmental remediation, and energy production either via the photovoltaic effect, or the photogeneration of energetic vectors.^3^, ^14^, ^73^

The success of heterogeneous photocatalysis is mainly related to the choice of inorganic semiconductors (mainly titanium dioxide, and transition metal oxides and sulfides) as photoactive materials, due to their ability to generate excitons after light absorption, which are able to provoke chemical transformation in other molecules (therefore, promoting the conversion of light into chemical reactions). Although much progress has been made, photocatalytic reactions still have numerous limitations to achieve satisfactory performance metrics with current photoactive materials based on semiconductors.^108^, ^109^

Despite the interest driven by carbon nanotubes and graphene in photochemical and optoelectronic applications,^19^, ^20^ and the structural and chemical similarities between amorphous carbons and graphene,^40^, ^41^ there has been a dearth in exploring the photochemical activity of these forms of carbon in the absence of a second photoactive phase (e.g., metals and semiconductors). Promising results have been reported in the fields of environmental remediation (i.e., wastewater treatment, self‐cleaning surfaces, and air purification), energy production by the photogeneration of energetic vectors (e.g., H_2_ and O_2_ from the photochemical water splitting and fuels from the photoreduction of CO_2_), and some other catalytic applications. However, some progress still needs to be done toward the understanding the fundamentals of the light/carbon interactions at a molecular level, their stability under long illumination periods (surface photocorrosion), or the improvement of the photochemical quantum yields of carbon materials. All this has opened up new perspectives for carbon materials in applied photochemistry (beyond their use as additives and supports to semiconductors) and thus deserves special attention.

The aim of this section is to provide an overview on the performance of carbon materials as photocatalysts in various fields of applications, covering the progress in advanced oxidation processes based on carbon photocatalysts for the degradation of pollutants and separation, energy production and conversion, as well as new possibilities of the photoactivity of amorphous nanoporous carbons in catalysis. Their role in semiconductor/carbon composites will not be considered here; it has widely been reported in the literature for all types of carbons and morphology as an alternative approach to improve the photoactivity of semiconductors, by the stabilization and separation of the excitons generated upon light absorption of the photoactive semiconductor in the π‐electron density of the carbon matrix.^12^, ^13^, ^15^

### 3.1 Photocatalytic Degradation of Pollutants

#### 3.1.1 Nanoporous Carbon Catalysts

The use of carbon materials in photocatalytic applications has been traditionally associated with their role as either additives or inert porous supports in semiconductor/carbon mixtures. The enhanced performance of the carbon/semiconductor composites had been exclusively discussed in terms of the enhanced mass transfer of the pollutant from the bulk solution due to the porosity of the carbon support, and/or to the superior mobility of the charge carriers within the graphitic sheets of the carbon nanostructure (predominant mechanism in CNTs or graphene/semiconductor composites).^12^, ^13^, ^26^, ^27^

The possibility of the photocatalytic activity of the carbon materials themselves however remained neglected for a long time. The studies of Luo et al.^21^ and Velasco et al.^28^, ^29^ reporting the formation of ROS upon irradiation of carbon nanotubes and nanoporous carbons in the presence of O_2_ or H_2_O, triggered the investigations of the photoactivity of semiconductor‐free nanoporous carbons as metal‐free and semiconductor‐free photocatalysts. Advanced oxidation processes (AOPs) for the degradation of pollutants (either in gas or in aqueous phase) appeared an interesting field to be explored.

**Figure 10:**
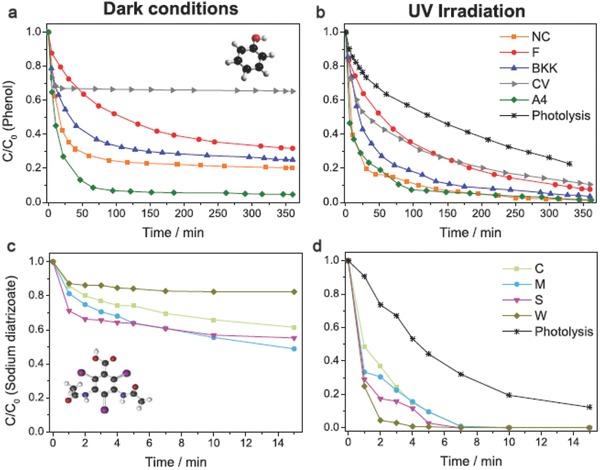
Concentration decay curves of phenol (a,b) and DTZ (c,d) on various nanoporous carbon photocatalysts under dark (a,c) and UV irradiation (b,d). Reproduced with permission.^102^ Copyright 2012, Elsevier. c,d) Reproduced with permission.^79^ Copyright 2017, Elsevier.

Velasco et al.^102^, ^110^ showed an improvement in the photo‐oxidation of phenol in an aqueous solution using nanoporous carbons as photocatalysts. Some carbons exhibited a higher photocatalytic activity for the oxidation of phenol than that of TiO_2_ powders; thus, the photocatalytic studies were extended to screening various nanoporous carbons obtained from different precursors (i.e., coal, plastic waste, lignocelluosic residues, and polymers) and showing varied chemical, textural, and structural features (**Figure** **10**). It was shown that the mechanism of photodegradation using nanoporous carbon catalysts differs from that reported for semiconductors. A marked regioselectivity for the formation of catechol over quinones was observed when nanoporous carbons were used as photocatalysts, as opposed to the preferential oxidation in ortho‐ and para‐position for n‐type semiconductors (such as TiO_2_, Bi_2_WO_6_, or WO_3_). This degradation pathway of phenol is more advantageous, as it involves a lower number of intermediate products; thus, eventually mineralization yields are high.^111^ Similar observations were also reported for semiconductor/carbon composites.^112^

Velo‐Gala et al.^53^, ^80^ reported the photocatalytic activity of various chemically modified commercial carbons for the degradation of sodium diatrizoate (an iodinated contrast and detected in urban wastewaters, surface waters, and ground waters) under UV and solar light (Figure 10). The photodegradation rate of sodium diatrizoate (excluding direct photolysis and adsorption) was enhanced in the presence of the nanoporous carbons. Such an improvement was found to be dependent on the amount of dissolved oxygen, chemical functionalization, and optical bandgap of the carbons.^53^, ^80^ The photocatalytic efficiency of the carbons under UV light was found to overcome that found for TiO_2_ under similar conditions, due to the different concentrations of ROS produced in both systems.

Impregnating the carbon with metals has also been reported to enhance the photocatalytic activity for the degradation of dyes and phenol. Sydorchuk et al.^113^ investigated the photoactivity of oxidized coconut shell–based activated carbons impregnated with copper and cobalt for the degradation of dyes (e.g., rhodamine B and methyl orange). The pristine carbon showed photocatalytic activity only under UV, as opposed to the cation‐exchanged carbons that were found to be photoactive under both UV and visible irradiation. For copper‐doped carbons, a similar increased effect was reported by Andrade et al. who attributed this behavior to the role of copper as an oxygen activator and the fast electron transfer environment provided by the metal.^112^

Seredych et al. described the photocatalytic activity of S‐ and P‐doped carbons and their composites with graphene toward the oxidation of dibenzothiophene (DBT) and its derivatives,^104^, ^114^, ^115^ and methylene blue.^92^ S‐and P‐doped carbons promoted the photodegradation of refractory sulfur compounds present in diesel, such as dibenzothiophene and dimethyl dibenzothiophene, to sulfoxides, sulfones, and other oxygen‐containing derivatives under UV and visible light. Oxygen from the carbon's surface groups and photogenerated holes and electrons was involved in the oxidation reactions. The presence of the graphene phase enhanced the transport of the charge carrier delaying their recombination, leading to the strong oxidative degradation of the refractory sulfur compounds.^104^, ^114^ S‐doped nanoporous carbons showed about twice higher photocatalytic activity for the degradation of methylene blue in simulated solar light than did commercial TiO_2_. The increase in the photocatalytic activity was attributed to the effect of sulfur on the optical features of the nanoporous carbon, allowing a better exploitation of sunlight. Further analysis of the factors affecting the photocatalytic oxidation of dibenzothiophenes on sulfur‐doped carbons showed that the oxidation was linked to the strong adsorption of the target compounds on the porosity of the studied carbons and to the enhanced dispersive interactions in small pores in the presence of sulfur.^115^

**Figure 11:**
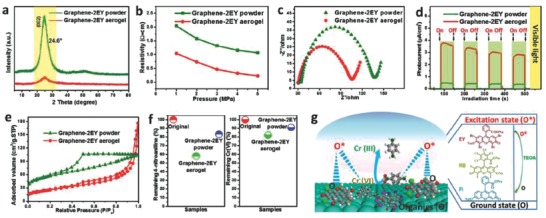
a) X‐ray diffraction (XRD) patterns; b) pressure–resistivity curves; c) electrochemical impedance spectroscopy (EIS) Nyquist plots; d) transient photocurrent spectra; e) N_2_ adsorption–desorption isotherms; and f) adsorption experiments of graphene–2EY aerogel and graphene–2EY powder. g) Schematic illustration of the proposed reaction mechanism for visible light–driven photocatalytic reduction of Cr(VI) over 3D graphene–EY aerogel photocatalysts. Note that O and O* in panel (g) represent the ground state and excitation state of the organic dye, respectively. EY represents modification with Eosin. Reproduced with permission.^116^ Copyright 2017, Elsevier.

Yang et al.^116^ studied on the activity of 3D metal‐free graphene–organic dye aerogels towards the reduction of Cr (VI) under visible light (**Figure** **11**). The authors reported the key role of the 3D‐interconnected porous structure of the graphene–dye aerogel in alleviating the aggregation of the structure, thus allowing an easy reutilization, as well as enhancing the adsorption of reactants and facilitating the photogenerated carriers to interact with the surface adsorbed reactants. The high electrical conductivity of the material (provided by the graphene‐like sheets) facilitated the spatial separation and rapid transportation of photoelectrons through the conductive graphene framework. Attaching the molecular dyes to the macroscopic 3D structure of the graphene aerogel integrated the ability of the carbon material to accumulate and transport the multielectron carriers generated by the excitation of the molecular dye attached to the surface. This allowed the efficient photosensitization mechanism.

Many commercially available nanoporous carbons present non‐negligible amounts of ashes (typically, inorganic mater in the form of oxides); thus, it is important to find out the effect of the ashes on the photocatalytic activity of nanoporous carbons. In this regard, Velasco et al. showed a decreased activity in the de‐ashed carbons toward phenol photo‐oxidation.^29^, ^30^ Even though contribution of the ashes to the photodegradation of the pollutant was not negligible, photoconversion of the de‐ashed carbons was higher than those corresponding to the photolytic degradation in the absence of a catalyst. On the other hand, the fact that the inorganic matter does not contribute to a decrease in the photocatalytic performance of nanoporous carbons constitutes an important finding from a technological viewpoint, since it allows us to facilitate the implementation of this materials in photocatalytic applications at a large scale, without the need to remove the ashes in an additional technological step.

**Figure 12:**
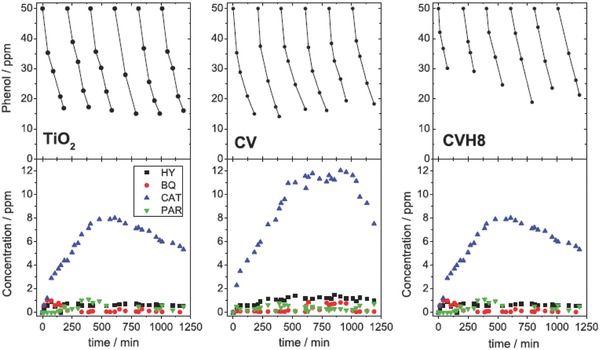
Evolution of phenol concentration (top) and its main degradation intermediates (bottom) after several consecutive photocatalytic cycles on nanoporous carbon CV and CVH, and TiO_2_ (P25, Evonik) under excess oxygen supply: hydroquinone (circles); benzoquinone (down triangles); catechol (squares); 2,4,6‐trihydroxybenzene (up triangles); resorcinol (crosses); 1,3,5‐trihydroxybenzene (diamonds). Reproduced with permission.^79^ Copyright, 2017, Elsevier.

With the purpose of evaluating whether or not nanoporous carbons as photocatalysts fulfill the requirements of long cycle‐life and good degradation efficiency, Velasco et al. investigated their performance during consecutive photodegradation runs,^117^ in comparison with a benchmark semiconductor (**Figure** **12**). For all the catalysts (carbons and titania), the performance decreased gradually with the number of cycles, due to the accumulation of degradation intermediates in solution (polyhydroxylated compounds and short‐alkyl‐chain organic acids), leading to poor mineralization degrees upon consecutive cycles. The cycleability of the nanoporous carbon photocatalysts was found to depend on their basic/acidic nature, with a somewhat lower performance of the hydrophilic carbons. The supply of dissolved oxygen was critical to assure a better long‐term performance over a large number of cycles, particularly in hydrophilic nanoporous carbon.

*Pollutant Nanoconfinement*: It should be pointed out that when porous catalysts are used, the reaction medium becomes complex due to the simultaneous occurrence of multiple phenomena: adsorption, photolysis (a noncatalyzed reaction), and photocatalysis. All of these processes affect the efficiency of the overall degradation reaction through different mechanisms, and might lead to biased interpretation of the photocatalytic activity of a porous catalyst. To isolate the contribution of the photochemical activity of the nanoporous carbons from secondary reactions and to discriminate the photochemical activity from the so‐called synergistic effect due to the porosity,^27^ Ania and co‐workers extended the photocatalytic studies to other nanoporous carbons following a novel approach.^63^, ^69^, ^102^, ^118^, ^119^ The approach was based on monitoring the photocatalytic reaction from a different viewpoint: inside the nanopores of the carbon photocatalysts. Briefly, the adsorption of the target pollutant (phenol was used as the reference pollutant) was allowed inside the pores of the carbon photocatalyst before irradiating the system and monitoring the photodegradation reaction of the molecules adsorbed inside the nanopores. The authors reported that for most of the investigated carbons, a significant fraction of the adsorbed phenol molecules was decomposed upon illumination, reaching a phenol conversion larger or similar than those in the noncatalyzed photolytic reaction. This is most remarkable considering the strong light‐shielding effect of the carbon matrix.^63^, ^69^, ^102^, ^118^, ^119^ The different photoactivity of the investigated carbons did not show a clear correlation with the specific surface area, pore volume, composition, or surface acidity/basicity, pointing out that the photochemical response of highly nanoporous carbons is also linked to the confinement state of a pollutant, and the structural and optical features of the carbon catalyst.

**Figure 13:**
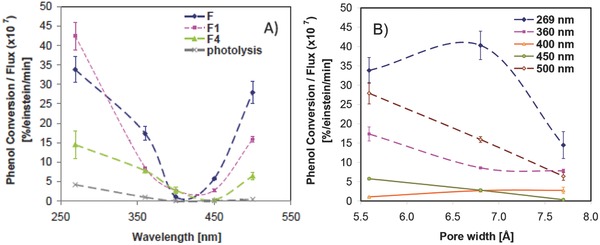
a) Phenol conversion per incident flux at different wavelengths on three nanoporous carbons. b) Dependence of the normalized phenol photo‐oxidation conversion on the average micropore size evaluated from gas adsorption data. Reproduced with permission.^69^ Copyright 2016, Elsevier.

The effect of nanoconfinement, surface functionalization, and wavelength of the irradiation source was investigated in a series of nanoporous carbons with controlled porous features using monochromatic light.^63^, ^69^, ^118^ Unlike polychromatic light, it allowed us to evaluate the impact of the energy of the incident photons on the photochemical conversion without biased interpretations. The porosity of the carbons was controlled by the activation of a nanoporous carbon of a narrow distribution of pore sizes in the microporous range using mild CO_2_ activation conditions. This treatment did not modify the surface chemistry of the carbons.^69^ Furthermore, the loading of the pollutant inside the pores was kept below the saturation limit of the carbons, in order to restrict the confinement of adsorbed molecules to the narrow microporosity of the nanoporous carbons. Phenol photoconversion levels inside the nanopores were higher than that of photolysis, indicating the key role of nanoconfinement in promoting light conversion into a chemical reaction (**Figure** **13**). Regarding the wavelength effect, most studied nanoporous carbons showed photocatalytic activity between 200 and 600 nm. The dependence of the performance of all carbons on the wavelength followed a U‐shaped pattern, with a minimum conversion between 400 and 450 nm. At the wavelengths corresponding to visible light, the conversion increased again with values very close to those obtained at 269 nm. The photochemical breakdown of phenol from solution under the studied conditions was negligible at wavelengths above 300 nm; in contrast, the onset of the phenol photo‐oxidation reaction in nanoporous carbons was measured at 432 nm, which corresponds to over 100 nm redshift in the wavelength onset in comparison to the noncatalyzed reaction.^118^ Moreover, high conversion values were obtained at 450 and 500 nm when the photochemical reaction was carried out inside the confined pore space.

The amounts of phenol oxidation intermediates (dihydroxylated benzenes) also varied with the confinement and illumination conditions.^69^, ^118^ At 269 nm both phenol and its aromatic intermediates were efficiently decomposed and under smaller energy irradiation the amount of intermediates gradually increased while the conversion of phenol did not follow the same trend. This pointed out some difficulties in pursuing the photo‐oxidation of dihydroxylated compounds under visible light.

Gomis‐Berenguer^63^ reported a wavelength dependence of the photo‐oxidation yields on the activation treatment. Despite the similar wavelength onset of the photochemical activity observed for all nanoporous carbons tested, a correlation between the variations in conversion and the confinement state of the adsorbed molecules was observed. A threshold pore size for an optimum exploitation of light depended on the dimensions of the target molecule retained in the porosity and on the strength of the interactions between the compound and the nanopore walls (adsorption forces). At 269 nm, phenol oxidation was enhanced in pores smaller than 0.7 nm, whereas the photochemical response drastically decreased when the pores were widened by 0.1 nm, demonstrating a more successful exploitation of light in tight confinements (Figure 13). For larger wavelengths, the drop in the photochemical conversion was very pronounced, particularly at 500 nm where the performance was quite close to that at 269 nm for pore apertures of 0.56 nm. This trend was attributed to the tight confinement of phenol molecules (molecular dimensions of phenol ≈0.80 × 0.67 × 0.15 nm) in the narrow micropores of the carbon, maximizing the host–guest interactions. When the pore aperture was enlarged, the interactions between the adsorbed phenol molecules and the pore walls became weaker,^120^ thus decreasing the probability of the fast charge transfer of the photogenerated carriers. In this situation, other electron donors and/or hole scavengers present in the medium (i.e., oxygen and water molecules) compete with adsorbed phenol molecules, resulting in lower conversions.

When methylene blue was adsorbed on the surface of a new generation of highly porous textiles,^121^ the dependence of photoactivity on the irradiation energy was also found. The surface of the textile was decorated with S‐, N‐, and O‐containing groups. Even though effects of the changes in the wavelength were very similar for all samples, the changes were mostly marked for textiles modified with nitrogen. Using a yellow filter resulted in the most efficient generation of photoelectrons, while the least efficiency was obtained upon using a red and a green filter. The results indicated the highest photosensitivity upon irradiation of about 500 nm. This is in agreement with the results published on the photodegradation of phenols where the sulfur doped porous carbons showed the best performance at visible light at 500 nm.^63^, ^69^, ^118^

As mentioned above, another important factor that controls the photocatalytic activity of nanoporous carbons is the chemical composition and, most importantly, the type and density of surface groups present on the carbon surface. Velasco et al. studied the effect of the incorporation of N‐ and O‐groups upon irradiation with polychromatic and monochromatic light.^118^ Oxidation of the carbon decreased the conversion of phenol and its intermediates due to the lower affinity of the pollutant to be adsorbed in oxidized carbons,^122^ pointing out the importance of the carbon–molecule interactions (physical and chemical) on the photocatalytic activity inside the pores. The oxidized carbons showed similar wavelength dependence than the pristine catalysts. Similar results on the effect of carbon oxidation were reported for the photocatalytic degradation of a pharmaceutical compound (i.e., citarabine), which presented lower affinity for oxidized carbons.^123^

N‐doping (mainly in the form of quaternary nitrogen and pyridine‐type groups) provoked a redshift in the light absorption, as evidenced by the increased photocatalytic conversion at 432 and 545 nm. These differences were explained in terms of the increased polarity and the electron transfer properties of carbon upon N‐functionalization due to electron‐donating character of the lone pair of electrons of the nitrogen atoms.^71^ It was proposed that the N‐groups would modify the density of electronic states of the carbon, by introducing intermediate states of higher energy to the band structure of the carbon materials^36^, ^124^ that would act as stepping stones facilitating the absorption of low‐energy photons.^118^

As in the case of O‐ and N‐groups, an improved photoactivity with an increase in the sulfur content at all wavelengths, particularly at 269 and 500 nm,^63^ toward the oxidation of phenol was found. A similar effect of S‐functionalization has also been reported for the photo‐electrochemical splitting of water using polychromatic light (see Section 3.2). The chemical environment of the S‐groups played an important role, with oxidized forms of sulfur being more photoactive than thiols and/or sulfides. The improved photocatalytic response after S‐functionalization was attributed to the narrowing of the average pore size, a fast charge transfer in S‐functionalized carbons and the stabilization of the holes through the oxidation of water adsorbed in the pores (as confirmed by electron spin resonance spectroscopic studies).^63^ Some other transitions involving the activation of chromophoric S‐groups under visible light were also proposed.

*Quantum Yield*: To gain additional insight into the photocatalytic activity of nanoporous carbons, a pseudo‐photochemical quantum yield (*Φ*_PS_) was estimated from the ratio of moles degraded per incident photon flux versus the irradiation time,^125^ and considering that the photon flux absorbed by the sample can be approximated to the photon flux emitted by the irradiation source (i.e., *I*_A_ ∼ *I*_0_).

(1) $$\Delta N=\phi I_{A}\Delta t$$

where *I*_A_ is the photon flux absorbed by the sample, evaluated from the product of the incident photon flux *I*_0_ provided by the lamp (determined by actinometry), and the integrated absorption fraction over the wavelength range used in the experiment.

**Figure 14:**
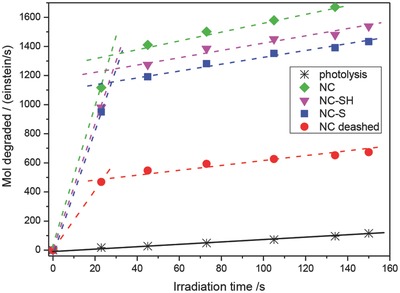
Pseudophotochemical quantum yield (φ_ps_) of a series of nanoporous carbons: NC, pristine carbon; NC‐S, carbon NC oxidized in ammonium persulfate; NC‐SH, sample NC–S treated at 400 °C under inert atmosphere; NC‐deashed, NC carbon after removal of ashes by acid digestion in HF/HCl. Reproduced with permission.^79^ Copyright 2017, Elsevier.

This approximation neglects the fraction of light absorbed by the carbon matrix (not used in the photocatalytic reaction), and the light scattering by the catalyst particles suspended in a solution. However, since the actual photon flux is certainly much lower than the incident flux of the lamp, the obtained value would account for the minimum limit of the actual quantum yield, allowing the comparison of the different carbons.^102^ The *Φ*_PS_ values obtained for various nanoporous carbons were higher than those corresponding to the photolysis of phenol in solution (**Figure** **14**). Furthermore, two different regimes were observed. Below 30 min of irradiation, *Φ*_PS_ values were close to unity, indicating a highly efficient photochemical reaction with almost one mole of a compound reacting per mole of absorbed photons. At longer illumination times, the slope of the response became smoother, rendering much lower *Φ*_PS_ values (although still about five times higher than photolysis). This indicated a fast consumption of the photogenerated charge carriers in the first stage (likely related to consumption of water and/or oxygen entrapped in the pores), leading to a diffusion‐limited rate in the second stage of the photodegradation reaction or a partial deactivation or consumption of some photoactive sites of the nanoporous carbon.

The oxidation of the carbon decreased promptly the *Φ*_PS_ values, indicating a sensitive response of the light conversion yield to small changes in the surface acidity of the nanoporous carbons. More specifically, the correlation with the nature of the O‐containing groups revealed that the impact of labile CO_2_‐evolving groups (attributed to carboxylic acids and anhydrides of acidic nature) was more pronounced than that of CO‐desorbing groups as quinones of phenolic moieties.^79^, ^119^

#### 3.1.2 Other Porous Carbon‐Based Materials

Based on the studies reporting the ability of CNTs to either generate reactive oxygen species under irradiation or to act as scavengers for ROS, Wu et al. have undertaken a study to clarify the predominating role of CNT in an aqueous environment in the photogeneration of reactive species.^85^ Using time‐resolved methods, the authors showed the CNTs' role as scavengers of the hydrated electrons formed upon high‐energy irradiation of the suspensions. In steady‐state UV irradiation, the nanotubes promoted the generation of ROS as singlet oxygen and hydroxyl radicals. Interestingly, the presence of CNTs suppressed the photodegradation of dye pollutants, as a result of the filter effect (i.e., light absorption effect) and the scavenging of the photogenerated ROS.^85^ The shielding effect of CNTs in semiconductor/CNT catalysts has been frequently reported, with most authors recommending low amounts of CNTs to avoid a decrease in the photocatalytic performance due to the absorbing and scattering of photons by the carbon matrix.^13^

### 3.2 Photoassisted Adsorption from Gas Phase

In spite of the development of new porous materials of exciting structure and chemistry, such as metal–organic frameworks^126^ or covalent organic frameworks,^127^ nanoporous carbons are still the adsorbents of choice in many applications involving separation processes. This is mainly due to their low costs and environmental inertness, for which new materials are still not found as being competitive.

One of the first separation applications which suggested the catalytic effects of sulfur, although no direct proof on the photoactivity had been provided, was the removal of toxic arsine from air.^128^ The experiments were done on the carbons derived from sulfur‐containing commodity polymers. To expand the range of surface properties, the carbons were further oxidized with ammonium persulfate or with air. The results collected indicated a very high catalytic activity of S‐containing carbons, which, in the presence of small amount of moisture from air, were able to oxidize AsH_3_ to As_2_O_5_ through a reaction in which a transfer of eight electrons is involved. Although the effect of a carbon surface photoactivity on the formation of OH radicals was not indicated, small amounts of water were found as beneficial for the oxidation process.

### 3.3 Photoenhanced Separation Processes

In many separation processes taking place on carbon surfaces, there is a demand to increase the specific adsorption forces to enhance the retention of target species on the surface, especially when there is a strong competition for adsorption sites between the compounds to be removed and a solvent. An example is desulfurization of liquid fuel where thiophene/benzopthiophenes have to be separated from the matrix of hydrocarbons, which contains, besides linear compounds, also aromatic ones such as naphtalenes.^129^ Nonmodified nanoporous carbons applied for this purpose are considered as rather nonselective adsorbents. To increase their selectivity for liquid fuel desulfurization, two approaches might be undertaken simultaneously. First, dibenzothiophenes need to be oxidized and then oxidation products should be adsorbed on the carbon surface. To impose the latter, the carbon surface must contain a significant amount of polar centers, which will specifically adsorb sulfoxides, sulfones, and sulfonic acids. Here sulfur‐containing carbon was found as beneficial adsorbents promoting both catalytic oxidation of dibenzothiophenes in visible light and their specific adsorption on the surface.^104^, ^114^, ^115^

First, the experiments were done on commercial wood‐based carbon and polymer‐derived carbons.^104^ Both were activated with phosphoric acid, and thus small amount of phosphorus was present in the matrix. The desulfization experiments from model diesel fuel were carried out in the dark, and under visible light and UV irradiations. Although both carbons showed some activity for DBT and 4, 6‐dimethyldibenzothophene (DMDBT) oxidation to sulfoxides, sulfones and other oxygen‐containing derivatives, the sulfur‐containing carbon was considered most active, and the formation of holes and electrons upon irradiation was suggested as responsible for the observed phenomena. The polymer‐derived carbon contained only 0.5 at% of sulfur in thiophenes, sulfides/thioesters, sulfoxides, sulfones, and sulfonic acids with the majority of thiophenes. The oxidized sulfur species were selectively adsorbed on the carbon surface, resulting in the clean fuel.

To further analyze the effect of sulfur incorporated into the carbon matrix on liquid fuel desulfurization, commercial wood‐based nanoporous carbon was treated at high temperature with hydrogen sulfide to introduce sulfur‐containing species.^115^ Functionalized carbons contentained ≈2.1 and 15 at% sulfur; the treatment also modified the texture of the carbons, with surface area values in the range of 2053 m^2^ g^−1^ for the pristine carbon, and 1722 and 1500 m^2^ g^−1^ after the incorporation of sulfur. The amounts of DBT and DMDBT adsorbed were found to be dependent on the sulfur content and the volume of pores similar in sizes to adsorbates' molecules. Exposure to either UV or visible light resulted in oxidation of both DBT and DMDBT to sulfoxides, sulfones, sulfonic acid, and other oxygen‐containing compounds formed as a result of the cleavage of C—C bonds in aromatic rings (**Table** **1**). The photoactivity was linked to the effect of sulfur, which, besides providing the energy gap, increased the ability of carbons to adsorb oxygen. Formed as a result of photoactivity, electrons reduced that oxygen to active superoxide ions. These species and holes contributed to the formation of OH radicals, provided that some water was retained in the pore system. Those species were, as indicated, responsible for the oxidation process.

**Table 1:**
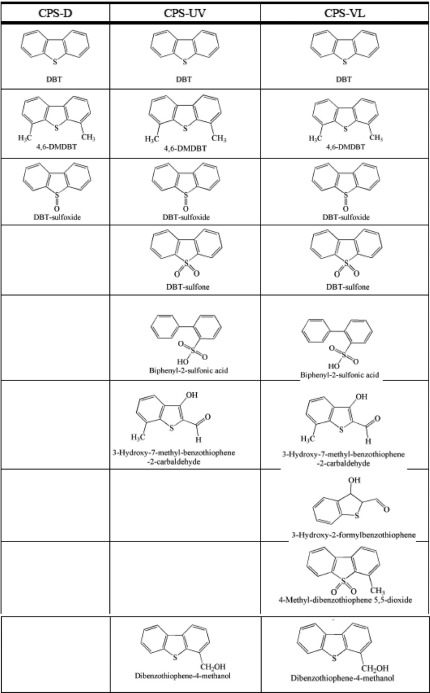
Speciation of the products detected in the extracts from the carbon samples' surface using a mass spectrometer (D, in the dark; UV, under UV light; VL, under visible light). Reproduced with permission.^130^ Copyright 2016, Wiley‐VCH

Since the effect of photoactivity on refractory sulfur compounds' separation is related to redox reactions, and for these processes the electron transfer process though the carbon matrix is considered as important (DBT and DMDBT are adsorbed in carbon pores), the effect of a graphene phase addition to S‐containing porous carbon on its photoactive desulfurization ability was investigated.^131^ As mentioned previously, the carbon was obtained from sulfur‐containing polymer, and 2 wt% of graphene was added to the polymer precursor to build the composite. This resulted in an about 40% decrease in the porosity and in an about four times increase in the DC conductivity and sulfur content (S increased from 0.4 to 1.6 at%). Reducing nature of graphene caused a significant part of S to be in thiophenic configurations, which were linked to photoactivity.

**Figure 15:**
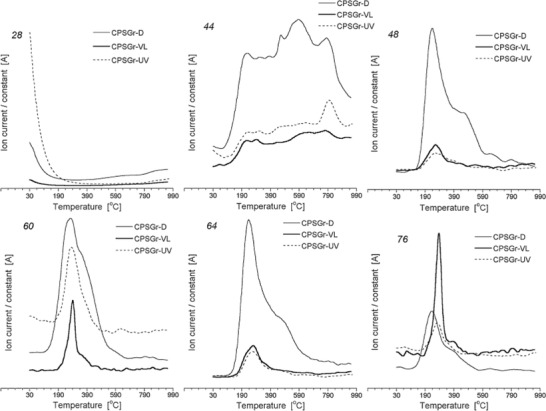
The mass spectrometer patterns for the composite material as obtained (denoted with letter D) and after exposure to UV and visible light. Assignment of fragments: *m*/*z* 28‐CO, *m*/*z* 44‐CO_2_, *m*/*z* 48‐SO, *m*/*z* 60‐CSO, *m*/*z* 64‐SO_2_, *m*/*z* 76‐CS_2_. Reproduced with permission.^141^ Copyright 2014, Elsevier.

Graphene phase also enhanced the transport of photogenerated electrons and increased the efficiency of an electron–hole separation. Holes were suggested as participating in the formation of OH radicals from water present in the pore system. It was also demonstrated that the photoelectrons formed as a result of photosensitivity reduced the surface of the carbon and the composite. The effect was stronger for the latter sample (**Figure** **15**). The results discussed above show the changes not only in the chemical nature of the adsorbed DBT and DMDBT but also in the nature of the carbon itself on which self‐reduction was found.

### 3.4 Energy Production and Storage

The generation of electricity in fuel cells from the electrochemical reaction of hydrogen and oxygen, coupled with photo‐electrochemical water splitting to produce oxygen and hydrogen gases from water, offers a viable approach to efficiently use sunlight.

#### 3.4.1 Oxygen Reduction Reaction

The slow kinetics of the oxygen reduction reaction (ORR) on the cathode limits the efficiency of a fuel cell and requires efficient and low‐cost catalysts. Nonmetal inorganic catalysts have been considered as the best options to eventually replace platinum, aiming to overcome the multiple drawbacks of Pt‐based electrodes, such as methanol cross‐over, CO deactivation, and high cost and scarcity.^132^ Metal‐free electrocatalysts based on carbon nanomaterials (e.g., graphene, carbon nanotubes, and graphitic carbon nitride) have revealed themselves as interesting materials of an electrocatalytic activity for the ORR, particularly those doped‐carbon nanostructures that show improved electronegativity, due to a synergetic charge transfer effect associated with the dopant and the favored O_2_ adsorption on the localized charged sites.^132^

Recent research efforts have led to the use of carbon‐based nanomaterial photocatalysts of 3D structures with the accelerated kinetics of the ORR process.^133^ Such a combination enables the utilization of photo‐ and electrochemical energy for applications in energy conversion in fuel cells. In this line, He et al. have reported the photoassisted enhancement of the activity toward the ORR of mesoporous g‐C_3_N_4_ under visible light. The superior transport properties of the photogenerated electrons in O‐ and S‐doped mesoporous g‐C_3_N_4_ were responsible for this behavior. Besides doping, the role of porosity seemed to be crucial as a small specific surface area usually renders a low performance.^134^

Some evidences have also been found for the reduction of oxygen in nanoporous carbons. Sing et al. suspected the photoassisted reduction of oxygen in S‐ and N‐containing nanoporous carbon electrodes exposed to visible light, based on the light‐driven enhanced capacity in acidic (sulfuric) medium.^135^ The increased capacitance upon illumination of the electrodes was linked to the oxidation of the carbon surface by photogenerated active radicals, which would make more pores accessible to the charge storage. The effect was not observed in a neutral electrolyte, or when oxygen was not present in the system.

#### 3.4.2 Water Splitting

Just like ORR is the key to the generation of electricity in fuel cells, the water splitting to produce oxygen (oxygen evolution reaction, OER) and hydrogen (hydrogen evolution reaction, HER) are the essence of metal−air batteries and electrochemical water‐splitting systems for a sustainable production of energy from renewable resources. From the application viewpoint, water splitting by electro‐ and/or photoassisted methods has been hampered by the low efficiency of the reaction—mainly due to the sluggish kinetics of oxygen evolution and the large overpotentials needed—as well as the scarcity and high cost of the precious metals used as catalysts. As examples, Pt‐based catalysts are the most efficient catalysts toward the ORR, while metal oxides (RuO_2_ and IrO_2_) are used for OER and HER.^136^, ^137^, ^138^ In practice, a photo‐electrochemical cell should integrate catalyst for both HER and OER, ideally in a shared aqueous solution. However, very often OER and HER catalysts are not stable under the same pH range. While most performing OER catalysts work well in neutral or basic media, most HER catalysts are only good in acidic media.

In the field of water splitting, despite carbon nanomaterials gained popularity in electrocatalysis as low cost and metal‐fee materials, their use as photoelectrodes has not been much explored, with all the studies focused on their role as photoanodes for the OER,^139^, ^140^, ^141^, ^142^ while nonporous carbon nanomaterials have been investigated for the HER.

**Figure 16:**
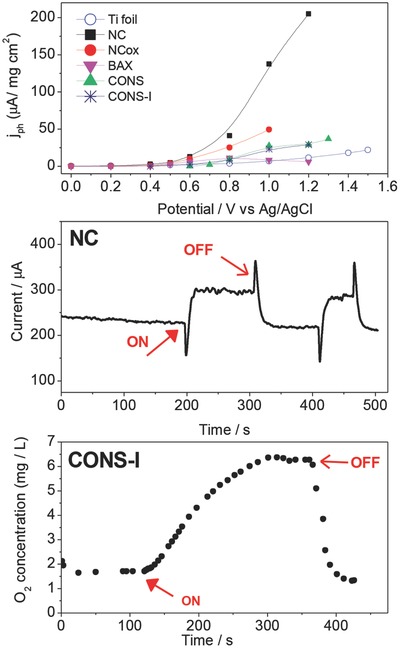
Top: Photocurrent versus bias potential of nanoporous carbons photoanodes exposed to simulated solar light; Middle: chronoamperometric response of carbon NC photoanode upon on/off illumination at +1 V versus Ag/AgCl, showing the square‐shaped profile and the cathodic/anodic shoots. Reproduced with permission.^79^ Copyright 2017, Elsevier. Bottom: Oxygen evolution during the chronoamperometric runs of sample CONS‐I. Reproduced with permission.^70^ Copyright 2014, Elsevier.

In this regard, and following the studies reporting the photocatalytic activity of nanoporous carbons for the oxidation of pollutants in aqueous phase (see Section 3.1) and their ability to generate radical species in water, their use as photoanodes for catalyzing the OER was explored.^70^, ^143^ For this purpose, low‐cost nanoporous carbons with varied chemical composition and textural features, to investigate the effect of heteroatoms (e.g., O‐, S‐, and N‐groups) and nanopore confinement, on their photocatalytic activity toward OER, were chosen. Carbon photoanodes prepared from organic polymer precursors showed remarkable photocurrent values at an overpotential of ≈0.8 V versus Ag/AgCl attributed to water oxidation (**Figure** **16**). This overpotential was clearly lower than that recorded for the naked current collector.^70^ The photocurrent response was stable and reproducible after several illumination cycles and upon several hours of illumination. The photocurrents were markedly higher than that recorded for a commercial wood‐based carbon used for comparison, with current densities up to 0.45 mA cm^−2^ for the highest potentials. For nonfunctionalized highly microporous carbon photoanodes, photocurrent values of ≈0.10–0.15 mA cm^−2^ were reported.^159^ Even if these values might not be among the highest reported for other water‐splitting photocatalysts, they were most outstanding for metal‐free nanoporous carbon photoanodes.

**Figure 17:**
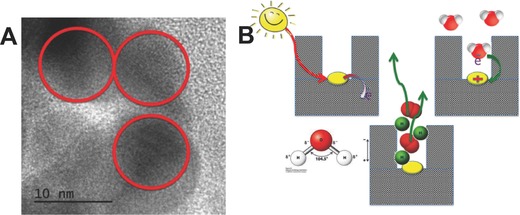
a) HRTEM of the nanoporous carbons obtained from S‐ and N‐containing polymers. The circles mark the sp^2^ carbon dots. b) Mechanism of water splitting on heteroatom‐containing nanoporous carbons. Chromophoric moieties are present in pores and they are excited by visible light. Electrons migrate through a carbon matrix, and holes are filled by electrons from the oxygen in water molecules. Reproduced with permission.^140^ Copyright 2016, Wiley‐VCH.

The ability of the carbons to oxidize water was linked to the presence of conductive graphene units and photosensitizers in these carbons, as well as to a well‐developed nanoporosity (**Figure** **17**). The hydrophobicity of the carbons is also important since it determines the wettability (rate and amount of water adsorbed). Regarding surface chemistry, light exposure caused the photogeneration of charge carriers on chromophore‐like moieties (e.g., sulfone/sulfoxides and some other O‐ and N‐containing groups) whose electrons were excited by irradiation leaving reactive vacancies (holes) able to accept electrons from oxygen in water molecules (in a similar way as reported for the visible‐light activity of some organic compounds).^70^

It is interesting to point out that the decoration of the carbon surface with O‐groups can also lead to a decrease in a photocatalytic response, depending on the nature of the groups.^143^ Oxidation of the carbons favors the surface recombination of the photogenerated charge carriers (i.e., lower photocurrents) due to the electron‐withdrawing effect of oxygen on the π‐electron density of the basal planes), which affects the stabilization/splitting of the exciton by a charge propagation through the carbon matrix. Additionally, the reduced photoactivity in oxidized carbons may be due to a lower density of free reactive sites in the edges of the carbon matrix (where the O‐groups are incorporated).

The Faradaic efficiency at short illumination periods was small, but increased upon irradiation reaching values close to 90%; this indicated that the charge was also consumed in side reactions. Further analysis of the anodes after illumination corroborated the photo‐electrocorrosion due to the occurrence of redox reactions involving the O‐, N‐, and S‐surface groups of the carbon matrix.^70^, ^139^, ^143^ Several complementary techniques confirmed the reduction of quaternary nitrogen, carboxylic acid groups, sulfonic acids, and sulfones, due to the side reaction of the photogenerated electrons with the carbon surface. This resulted in the consumption of the photoactive sites of the nanoporous carbons and in charge transfer limitations. The strong surface reducing effect upon light exposure was linked to the affinity of the carbons to adsorb water, as an essential part of the efficient electron transfer mechanism to the chromophore groups of the carbons.

#### 3.4.3 Light‐Enhanced Capacitive Behavior

During recent decades, a significant stress in the energy‐related research has been placed on carbon‐based supercapacitors.^42^, ^43^, ^144^ For this application, to reach a high storage capacity, and high power and energy densities, a combination of various factors is needed. It includes the carbon porosity, chemistry, conductivity, or electrochemical stability. They are important for electric double layer capacitance (EDLC), pseudocapacitance, charge transfer, or the applied voltage range, respectively.

**Figure 18:**
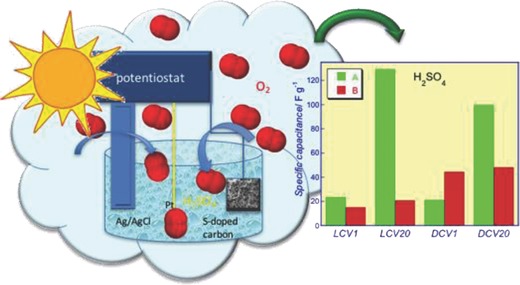
Specific capacitance values obtained under the different experimental conditions in H_2_SO_4_. (L, light; D, dark; CV1, the values calculated from the first CV run; CV20, the value calculated from the 20th CV run; A, electrolyte saturated with air; B, electrolyte purged with nitrogen. Reproduced with permission.^141^ Copyright 2014, Elsevier.

Even though the photosensitivity of nanoporous carbons is not of crucial importance for the charge storage applications, there are recent reports addressing an increase in capacitance upon irradiation. That effect was found when sulfur‐doped carbon (3.3 at% S) of a high density and small porosity was tested as a supercapacitor in acidic or neutral media.^135^ The sulfur species, besides contributing to pseudocapacitance,^145^ were suggested as contributing to ORR. Superoxide and/or H_2_O_2_ formed in this reaction oxidized the surface of carbon during a cycling. This resulted in the formation of more pore volume for EDLC. Since the photosensitivity led also to the formation of holes and electrons, the latter increased the efficiency of ORR and also limited the extent of surface oxidation. Photogenerated holes, on the other hand, were suggested as participating in the formation of active radicals/oxidation of water which resulted in more oxygen in the system and the regeneration of H^+^ in an acidic electrolyte. After 20 cycles of the cyclic voltammetry experiments, an increase in the capacitance was observed owing to the photoactivity and involvement of oxygen, either that dissolved in an electrolyte or formed from the reduction of sulfur species or water oxidation. Differences in chemical nature of electrolytes contributed to the complexity of surface processes. Their effect on capacitance is visualized in **Figure** **18**, where a marked increase in the capacitance was found under visible light irradiation LCV20) with oxygen in the electrolyte (condition A).

This complex effect of photosensitivity on the capacitive performance was further explored on S‐ and N‐doped nanoporous carbon in an acidic electrolyte.^131^ The contents of sulfur in C–A, C–B, C–AO, and C–BO was 1.7, 2.4, 0.7, and 0.9 at%, respectively. The measured capacitance reached 450 F g^−1^ in spite of a relatively low surface area. The results showed a small but consistent increase in the capacitance after visible light irradiation. Thus, to test the photosensitivity, chronoamperometry experiments were run at constant bias potential values in dark and under visible light. The latter visibly increased the cathodic current measured.^131^ Since this effect was not observed in the absence of oxygen, it was linked it to oxygen reduction process on the surface of the electrodes.^146^, ^147^ After wetting in water, the order of a decreasing photocurrent was C–A > C–B > C–AO = C–BO and sulfuric acid wetting changed the order to C–B > C–AO = C–BO > C–A. Neutral electrolyte wetting obviously showed the C–A sample as the one with the most photoactive surface, and it was linked to the high content of sulfonic acids/sulfoxides and sulfones in the electrode. The generation of photocurrent was linked to the excitation of sulfonic/sulfoxide chromophore‐like moieties decorating the surface of the polymer‐derived carbons. Moreover, the carbon tested had graphitic dots of ≈10 nm in their microstructure which were linked not only to the enhanced conductivity but also to the source of its energy bandgap/photoactivity, as suggested by Robertson and O'Reilly.^35^, ^36^

#### 3.4.4 Light‐Enhanced CO_2_ Reduction

The photochemical conversion of CO_2_ into fuels and other compounds of industrial interest (e.g., CO, CH_4_, CH_3_OH, and HCOOH) has also been widely explored, driven by the need to manage and transform the increasing amounts of carbon dioxide emitted to the atmosphere. Among various methods, the photocatalytic reduction of CO_2_ with H_2_O is regarded as one of the most promising and yet challenging approaches.^148^, ^149^, ^150^

Recently Li et at.^59^, ^151^ reported the changes in the surface features of S‐and N‐doped carbons obtained from a polymeric precursor upon the exposure to light with water or/and CO_2_ preadsorbed on the surface. The results showed that in all cases the surface chemistry was affected, some species got oxidized and removed (thiophenic sulfur in carbon) and also changes in the carbon's porosity were noticed as a result of light‐induced reactivity of water and CO_2_ with the carbon surface. The clear effect of water decomposition was found upon irradiation.

For comparative purposes, a physical mixture of the S‐doped carbon and the polymer, g‐C_3_N_4_, used as a N‐source was also investigated.^59^ Although it was found to be more active in water splitting and CO_2_ reduction than the carbon itself, it underwent self‐reduction owing to a low conductivity. The most reactive conditions for the physical mixture were those under light with CO_2_ and water in the system, where water and CO_2_ could undergo surface reactions. In the case of the composite, photoactivity, reactivity and formation of oxygen resulted in the oxidation of the carbon matrix, which also led to a porosity decrease. Even though upon light exposure water splitting on the surface of the photocatalysts took place, both water splitting and CO_2_ reduction were much more favorable on the physical mixture of the S‐doped carbon and the N‐dopant. Its bandgap was 2.1 eV, and the positions of VB and CB (potentials) were found to be most favorable for water oxidation and reduction, and CO_2_ reduction. The densities of both holes and electrons were in the order of 10^−13^–10^−14^ cm^3^.

Since the results suggested that CO_2_ can undergo reduction upon exposure of the carbon to light, these conditions were also tested in photo‐electrochemical environment.^152^ For it, commercial carbons were modified by thiourea at moderate (600 °C) and high (950 °C) temperatures. The results showed an increase in the Faradaic efficiency of the CO production owing to the photoactivity. It was suggested that thiophenic‐S increases carbon photosensitivity and promotes CO formation. This was supported by the bandgap energy and band alignments. The photoactive catalyst should have a bandgap of less than 3.18 eV in order to adsorb visible light. BAX‐TU‐600 and BAX‐TU‐950 show bandgaps of 1.91 and 3.02 eV, respectively. The smaller bandgap of BAX‐TU‐600 in comparison to that of BAX‐TU‐950 was linked its higher photoactivity. The position of the CB of BAX‐TU‐600 was more favorable than that of BAX‐TU‐950 for both the CO_2_ reduction and H_2_ evolution reactions, which is consistent with the higher extent of photocurrent generation at light on BAX‐TU‐600 as compared to that on BAX‐TU‐950.

### 3.5 Other Photocatalytic Reactions

In the context of looking for alternatives to expensive noble metal catalysts, there has been much interest in exploring the possibilities of carbon materials as metal‐free and semiconductor‐free catalysts. Indeed, there is ample literature data showing that different defective, doped and functionalized forms of carbons can exhibit a high catalytic activity for a variety of reactions competing with metal‐based conventional catalysts.^153^, ^154^, ^155^, ^156^

**Figure 19:**
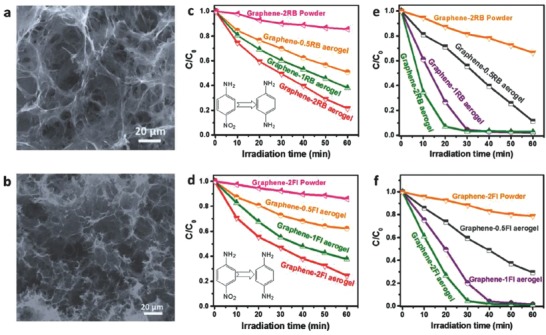
SEM images of a) graphene–RB aerogel and b) graphene–FI aerogel. Visible light‐driven (*k* > 420 nm) photocatalytic hydrogenation of 4‐NA to 4‐PDA over c) graphene–RB aerogel and d) graphene–FI aerogel, and reduction of Cr(VI) over e) graphene–RB aerogel and f) graphene–FI aerogel. RB represents Rose Bengal, FI‐fluorescein, NA‐nitroaniline, and PDA‐phenylenediamine. Reproduced with permission.^116^ Copyright 2017, Elsevier.

Yang et al.^116^ reported the activity of 3D metal‐free graphene–organic dye aerogels toward the photocatalytic hydrogenation of nitro compounds under visible light (**Figure** **19**). The excellent photocatalytic activity of the material for reduction reactions was attributed to the fast transportation of photoelectrons through the conductive graphene framework, and the macroscopic 3D structure that prevented aggregation of the catalyst and enhanced the adsorption of reactants. Furthermore, attaching molecular dyes on the surface of the catalyst allowed an efficient photosensitization mechanism by the excitation of the molecular dye.

### 3.6 Photoluminescence

**Figure 20:**
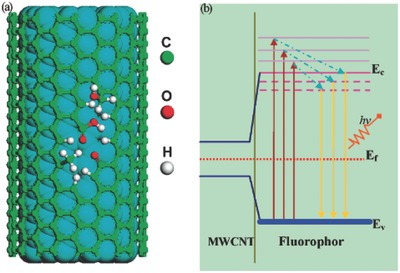
a) Schematic model shows functional groups absorbed at the defects are of multiwalled CNTs. b) Schematic diagram of the mechanism for PL (the left part corresponds to the inner layers of multiwalled CNTs with perfect structure; the right pat corresponds to the outmost layers with high density of defects and functional groups). Reproduced with permission.^180^ Copyright 2007, ACS Publications.

Photoluminescence is the optical property of matter, which represents emission after the absorption of photons. It is initiated by photoexcitation. In the category of carbon‐based materials, the photoluminescence phenomenon was first studied on carbon nanotubes.^157^, ^158^ It is generally accepted that photoluminescence in CNTs is linked to the presence of defects in the form of chemical group. Bao et al. referred to them as fluorophores,^159^ and their density functional theory (DFT) calculations showed that these groups changed the electronic structure of CNTs by opening the bandgap, and in this way affected the transitions between the excited state and the equilibrium state (**Figure** **20**).

Discovery of graphene further advanced the study of photoluminescence of carbon‐based materials in the context of exciting applications of graphene/carbon quantum dots (CQDs) in bioimaging,^160^, ^161^, ^162^ drug delivery,^163^ photocatalysis,^164^, ^165^ electrocatalysis,^165^ photovoltaics,^166^, ^167^ and sensing.^168^ Those CQDs^169^ are a new form of fluorescent carbon nanostructures. Their diameters are smaller than 10 nm, and they are considered superior to conventional heavy‐metal semiconductor quantum dots owing to the low toxicity, high biocompatibility, water solubility, optical stability, easiness of functionalization/modification, and low cost. Their photoluminescence characteristics are linked to the sizes and ratio of sp^2^ and sp^3^ domains. The mechanism is considered complex and involves 1) defects' state emission/surface energy traps and 2) intrinsic state emissions, which include electron–hole recombination, quantum size effects/zigzag states.^170^, ^171^, ^172^, ^173^, ^174^ The emission characteristics were linked to surface chemistry and thus to alterations in the bandgap.^175^ The emissive traps on the surface were also found to be affected by ions or solvent molecules attached to the surface of CQDs. It has been proposed that by changing surface chemistry the mechanism of photoluminescence changes. Thus, amination transfers carboxyl and epoxy groups to —CONHR and —CNHR that reduce nonradiative recombination and thus result in intrinsic state emission. Reduction of the surface transfers those carboxylic and epoxy groups into OH moieties which also decrease the nonradiative process by reduction of the defects and enhancing the integrity of π‐conjugated systems. Those CQD, owing to their relatively simple structure, were indicated by Zhu et al.^175^ as model materials leading to understanding the photoluminescence mechanism of carbon‐based materials.

There are limited studies on the photoluminescence of nanoporous carbons. This might be related to the complexity of their surface. Another reason is that, and up to now, the applications of this kind of materials in optics or optoelectronic have rather not been explored. Baranauskas et al.^98^ studied the photoluminescence properties of porous carbon films obtained from polyvinyl alcohol by its pyrolysis at various temperatures (333–843 K). The films were deposited on silicon and porous silicon surfaces. The carbon phase was considered as macroporous and had the characteristics of both glassy and diamond‐like carbon, based on Raman spectra. Hydroxyl groups were also detected on the surface. The luminescence intensity depended on the pyrolysis temperature. Thus, the sample pyrolyzed at 333 K showed little luminescence, and the strongest effects were found for the films synthesized at 513 and 623 K. A further increase in the pyrolysis temperature resulted in weaker signal. This trend was explained by the presence of hydrogenated carbon structures in the films pyrolyzed at 513 and 623 K. It was suggested that a strong luminescence was the result of the activation of the hydrogenated structures of the crystalline phases of the carbon films. The high synthesis temperatures decreased the hydrogen content and thus the intensity of luminescence peaks.

For the first time, the photoluminescence of nanoporous was studied on the polymer‐derived materials.^176^ The carbon contained oxygen, sulfur, and nitrogen groups, and was rich in graphitic domains with sizes of about 10 nm. The photoluminescence in the visible range was recorded, in spite of a strong light‐absorbing nature of carbon matrix. The difference in the intensity of the signal and the luminescence range was linked to the differences in surface chemistry and especially to the presence of sulfones, sulfoxides, sulfonic acids, and nitrogen moiety, which act as chromophores in the carbon pore system. On these species, upon absorbing radiation close to visible range, electrons were transferred to the graphitic domains of carbons matrices, which were in the vicinity of these groups. This process led to the photoluminescence phenomenon. It was also found that adsorption of nonpolar species in the carbon pores quenched the photoluminescence intensity. This behavior suggested that such materials could be used in optical sensing devices.

## 4 Foreseen Applications

### 4.1 Photovoltaics

In photovoltaic applications, nanoporous carbon materials have been investigated as counter electrodes in dye‐sensitized solar cells (DSSC). In general, nanoforms of carbons and carbon nanotubes have shown a good catalytic activity for the I_3_^−^/I^−^ redox reaction, showing a remarkable current density improvement and solar power conversion efficiency of around 8%.^177^, ^178^ These values might be lower than those reported for DSSC based on Pt‐counter electrodes,^179^, ^180^ but they were obtained with metal‐free nanoporous carbons as counter electrodes.

Since the conductive nature of carbons is an asset for electron‐transfer processes, there are reports in the literature addressing graphene and graphene oxide,^180^ carbon nanotubes,^181^ and CQDs^167^, ^182^, ^183^, ^184^ as electrodes for dye‐sensitized solar cells^185^, ^186^ and perovskite solar cells.^187^ Moreover, graphene‐based solar cells are also the subjects of photovoltaic studies.^188^ To improve the efficiency of DSSCs, a wide‐bandgap semiconductor is needed that would be able to adsorb active dyes and transport the injected electrons into an electrical circuit. Taking this into account, photoelectrodes of a DSSC should be porous (mesoporous) to adsorb large dye molecules. A goal here is a high density of dyes and minimization of the resistance to electrolyte diffusion to the adsorbed dye molecules.

The most common electrodes are those based on titania. Even though the CQDs, graphene oxide, or CNTs are not considered as highly porous, they provide conductivity and charge mobility. This combination increases the efficiency of cells with the modified photoelectrodes of DSSCs, which is caused a quick transport of electrons from the excited dye molecules into the conduction band of TiO_2_ through the carbon phase. When too much of carbon phase is used, the incident photon‐to‐current efficiency decreases due to highly light‐absorbing properties of the carbons phase.

Batmunkh et al. have discussed the methods of graphene modifications to improve its interactions with dyes.^185^, ^186^, ^187^ It has been found that chemical treatment of graphene improves its interactions with dyes and has an effect on the size of the bandgap. Sun et al. found that addition of only 0.5 wt% graphene to TiO_2_ increased the conversion efficiency about one order of magnitude, due to an enhanced dye adsorption and a significantly longer electron lifetime.^189^ Besides graphene, CNTs, or CQDs, carbon fibers were also used for this purpose.^190^ Building such a device based on the latter, besides the increase in the efficiency, rendered flexibility to the system.

Interesting results have also been obtained for biomass‐derived carbon nanostructures used as counter electrodes.^177^, ^178^ Lowpa et al.^177^ reported a 7.71% efficiency on a DSSC based on nanoporous carbon microspheres, which was close to that obtained for Pt‐DSSC (≈8.05%). The result was interpreted in terms of the decrease in a polarization resistance at a counter electrode interface when the porous carbon was used. The nanoporous carbon microspheres improved their performance after annealing for the I_3_^−^/I^−^ redox reaction, with peak currents almost identical to that of Pt electrode, except for a negative voltage shift of the reduction peak potential from 0.15 V (Pt) to 0.03 V in the carbon counter electrode. This suggested that the carbon microspheres can supply electrons to oxidizing agents easier than does Pt.

Xu et al.^178^ reported the use of biowaste‐derived carbons as counter electrodes, using biomass from different origins. Although the DSSC based on biowaste‐derived carbon counter electrodes showed higher efficiency than graphite electrodes, the values were still far from those obtained for Pt‐electrodes, ranging between 0.77% and 4.70 %. Interestingly, the highest efficiency was measured on carbons obtained from woods and leaves; this was attributed to the superior catalytic activity and a faster electron transport for the I_3_^−^ reduction occurring in the unique morphological and structural features of those carbons. They had a hierarchical fibrous carbon skeleton at a nanometric and micrometric scale, with abundant exposed edges and defects, and oxygen‐containing surface groups. In all cases, the performance of the photovoltaic devices was discussed in terms of an enhanced conductivity of the carbon counter electrode and the fast I_3_^−/^I^−^ reduction. So far, the contribution of the photoactivity of the carbon material to the efficiency of the DSSC has been disregarded.

The application of the porous carbon for DSSC was reported by Wu and co‐workers.^191^ In their devices, well‐ordered mesoporous carbons, activated carbon, carbon nanotubes, carbon black, conductive carbon, carbon dye, carbon fibers, tonner printer, and fullerenes were used as additives to TiO_2_. The carbons had the surface areas ranging from 385 to 721 m^2^ g^−1^. Even though all carbon materials tested showed a catalytic activity for triiodide reduction to iodide, the mesoporous carbon exhibited the performance similar to that of a Pt counter electrode. Although the trends in the effects of the surface area on the performance have not been discussed by Wu and co‐workers, the results indicated that a limiting diffusion current density depended on the diffusion coefficient of the triiodide species in the electrolyte, and for this the porosity might be an important factor. In fact, the ordered mesoporous structure (*S*_BET_ = 659 m^2^ g^−1^) was indicated as beneficial for the redox couple diffusion in the electrolyte. The conversion efficiencies measured with the porous carbon counter electrodes were between 2.8% and 7.5%. With the ordered mesoporous carbon the efficiency was 7.7% and using of the commercial activated carbon resulted in the efficiency of 6.5%.

Graphene‐based Schottky junction solar cells are another type of solar cell containing a carbon phase, which have been recently explored.^192^ Even though their conversion efficiency is still low (a few percentages) they are considered as advantageous compared to those based on indium tin oxide (ITO)–Si junctions. It is because of the broad range of possibility of tuning graphene work function, its superior electrical and optical properties, and, last but not the least, the cost factor. It has been reported that chemical doping of graphene with bis(triofluoromethanesulfpnyl) amide increased the performance of undoped device over four times.^188^ An increase in the efficiency was linked to the shift in the graphene chemical potentials and to an increase in the carrier density.

### 4.2 Photonic Crystals

In the context of photonic applications, porous carbon colloidal photonic crystals (black opals) have been synthesized by nanocasting procedures in a centimeter range length.^193^ Despite the black color of the carbon opal, the material exhibited a clear opalescence changing with the observation angle. Although the photonic properties of the carbon opal are related to the periodicity of its 3D structure, rather than to the optical features of the carbon matrix itself, the interest lies in the possibility of synthesizing such structures using low‐cost materials.

### 4.3 Inhibition of Photocorrosion

Mitigating photocorrosion in a light‐absorbing material is a subject of major research in various disciplines (e.g., photo‐electrochemical solar water splitting and metals protection). The use of stable protective layers has been explored, mainly based on semiconductors. Some carbon materials have also been investigated for this purpose, including graphene, g‐C_3_N_4_, and their composites with other materials. A few works have reported the protection of metal oxides photocathode by graphene, TiO_2_/graphene, and TiO_2_/CNT coatings.^194^, ^195^, ^196^, ^197^ The stability of the coated photocathodes significantly increased, particular for the composite, due to the highly conductive nature of CNTs, the separation of the charge carriers, and the suitable band alignment of the protective TiO_2_/graphene layers. TiO_2_/CNT coating films exhibited three times higher photocurrent and half the charge transfer resistance of pure TiO_2_ films, and provided a much better photocathode protection for steel under UV irradiation.^197^

## 5 Concluding Remarks

In many photoactivity studies of nanoporous carbon materials reported in this review, an inspiration was in their semiconducting nature and in similarity to graphene, whose distorted and full of defects units are, in fact, the building blocks of their pore walls. Of course, their unique and important aspect is a high porosity for which extent only metal organic framework (MOF) of covalent organic framework (COF) can be considered as being competitive. As indicated in the “Introduction,” the studies on the photoactivity of nanoporous carbons are still in their infancy. Nevertheless, we have shown that in some applications these materials can be considered as advantageous as are other nanoforms of carbons. Examples are in advanced oxidation processes, separation processes, photocatalysis, or even DSSC applications. For instance, the progress beyond a current state‐of‐the‐art in advanced oxidation processes based on nanoporous carbon photocatalysts for the degradation of pollutants relies on simultaneously promoting the in situ photodegradation of pollutants and regeneration of the exhausted carbon bed. Graphene, carbon nanotubes, or even CQDs have recently been extensively studied from the viewpoint of their optical and electronic properties. That abundance of reports, and only a small fraction of them, has been addressed here, might be not only due to exciting properties but also due to a relatively simple structure and chemistry of CNT or graphene. Their simplicity is naturally an asset in understanding and in explaining new phenomena. Nanoporous carbons, on the other hand, are complex. That complexity is not only in their amorphous nature and but also in impossibility of the separation of the effects of their chemistry, microstructure, and nanopore confinement. We have shown here that these materials have all the features, which were indicated as governing the photoactivity of graphene including small sp^2^ units, sp^3^ configurations, and abundance of surface defects. Moreover, besides heteroatoms doped to their distorted graphene matrix, their surface can also be decorated with S‐, N‐, O‐, and P‐containing moieties, which can act as chromophores or even as pseudo light‐sensitive dyes attached to the conductive matrix. Even though nanoporous carbons are one of the oldest materials, in our opinion, owing to their unique properties, they deserve another look and reaching beyond adsorption in their applications. Perhaps the future of photovoltaics, smart‐self‐cleaning surfaces and photocorrosion protection, or solar energy conversion and CO_2_ mitigation, is in exploring the use of nanoporous carbons. The challenge is of course in their complexity. We hope this review will help not only to take another look at nanoporous carbons, but will also help to stimulate research leading to better understanding their exciting behavior, which, even few years ago, was not considered as having any physical bases. Examples are in the statement often encountered by us in the past in scientific discussions concerning the photoactivity of nanoporous carbons: “carbons do not have a bandgap …”.

## Conflict of Interest

The authors declare no conflict of interest.
